# Does an Integrated Care Intervention for COPD Patients Have Long-Term Effects on Quality of Life and Patient Activation? A Prospective, Open, Controlled Single-Center Intervention Study

**DOI:** 10.1371/journal.pone.0167887

**Published:** 2017-01-06

**Authors:** Elena Titova, Øyvind Salvesen, Signe Berit Bentsen, Synnøve Sunde, Sigurd Steinshamn, Anne Hildur Henriksen

**Affiliations:** 1 Department of Circulation and Medical Imaging, Norwegian University of Science and Technology, Trondheim, Norway; 2 Department of Thoracic and Occupational Medicine, Trondheim University Hospital, Trondheim, Norway; 3 The Faculty of medicine, Norwegian University of Science and Technology, Trondheim, Norway; 4 Department of Health Education, University of Stavanger, Stavanger, Norway; Dartmouth College, UNITED STATES

## Abstract

**Background:**

Implementation of the COPD-Home integrated disease management (IDM) intervention at discharge after hospitalizations for acute exacerbations of COPD (AECOPD) led to reduced hospital utilization during the following 24 months compared to the year prior to study start.

**Aims:**

To analyze the impact of the COPD-Home IDM intervention on health related quality of life, symptoms of anxiety and depression, and the degree of patient activation during 24 months of follow-up and to assess the association between these outcomes.

**Methods:**

A single center, prospective, open, controlled clinical study. Changes in The St. George Respiratory Questionnaire (SGRQ), the Hospital anxiety (HADS-A) and depression (HADS-D) and the patient activation measure (PAM) scores were compared between the patients in the integrated care group (ICG) and the usual care group (UCG) 6, 12 and 24 months after enrolment.

**Results:**

The questionnaire response rate was 80–96%. There were no statistically significant differences in the change of the SGRQ scores between the groups during follow up. After 12 months of follow-up there was a trend towards a reduction in the mean HADS–A score in the ICG compared to the UCG. The HADS-D scores remained stable in the ICG compared with an increasing trend in the UCG. Clinically significant difference in the PAM score was achieved only in the ICG, 6.7 (CI95% 0.7 to 7.5) compared to 3.6 (CI95% -1.4 to 8.6) in the UCG. In a logistic regression model a higher HADS-D score and current smoking significantly increased the odds for a low PAM score.

**Conclusion:**

The COPD–Home IDM intervention did not result in any statistically significant changes in mean SGRQ, HADS-A, HADS- D or PAM scores during the 24 months of follow-up.

**Trial registration:**

The ID number for the study in the Clinical.Trials.gov registration system is 17417. ClinicalTrials.gov Identifier: NCT 00702078

## Introduction

Chronic obstructive pulmonary disease (COPD) is a leading cause of morbidity and mortality worldwide and represents an increasing economic and social burden [1]. The optimal care for patients with COPD requires an individualized, patient-centered approach that recognizes and treats all aspects of the disease, addresses the systemic effects and co morbidities, and integrate medical care among healthcare professionals and across levels of healthcare [2]. The concept of integrated care, the specific components and its potential in the management of COPD patients has been described previously [3].

A Cochrane review from 2013 of integrated disease management (IDM) interventions among COPD patients showed that IDM not only improved disease–specific quality of life and exercise capacity, but also reduced the number of hospital admissions and in-hospital days per person [4].

However, more recent publications on IDM among patients with COPD have shown controversial results. Hernandez and co-authors [5, 6] found that IDM improved clinical outcomes such as survival and the number of emergency department visits, but not the number of hospital admissions. In the study by Kruis et al [7] no additional benefit of IDM compared to usual care was found, except an improved level of integrated care and a higher degree of self-reported daily activities.

It is debated why IDM interventions are successful in some patients and a failure in others. In part the variation may be explained by differences in essential preconditions such as the patients`beforehand knowledge and confidence when given the role as managers of their own health [8]. The success of an IDM intervention is by large dependent on to what degree the participants become involved in their own healthcare, but little is known regarding which basic qualities are required among the often old and multi-morbid COPD patients to become motivated for such changes. One approach may be to estimate patient activation which refers to people’s ability and willingness to take on the role of managing their own health and healthcare [9, 10]. Previous research suggests that the degree of patient activation is a factor that may predict emergency room and hospital utilization as well as adherence to medication in chronically ill patients [9–11]. However, knowledge regarding the association between patient–activation and the success of IDM interventions among COPD patients is lacking.

Previously we have shown that when implementing the COPD-Home IDM intervention [12] at discharge after AECOPD related hospitalizations, the number of hospital admissions were reduced during the 24 months of follow–up compared to the year prior to study start [13].

The aims of the present study were to examine the impact of the COPD-Home IDM intervention on health related QOL, symptoms of anxiety and depression and the degree of patient activation during 24 months of follow-up and to assess the degree of association between these outcomes.

## Materials and Methods

### Study design and setting

A prospective, open, controlled single-center intervention study with four repeated measurements was conducted at Trondheim University Hospital (TUH) and in Trondheim municipality between March 2008 and November 2011.

#### Participants

All patients, who were hospitalized at the Department of Thoracic Medicine (DTM) or at the Observation Unit of TUH and who fulfilled the inclusion criteria, received information about the COPD-Home study and were invited to participate. The inclusion criteria were: (a) admission due to AECOPD, (b) COPD stage III or IV (GOLD 2007), (c) living in the Trondheim municipality, (d) an ability to communicate in Norwegian, and (e) an ability to sign the informed consent form. The exclusion criterion was: affected by any serious disease that might cause a very short lifespan (expected survival time less than six months). Patients, who gave their consent to participate, were consecutively enrolled in the study before discharge from the TUH. They were randomly allocated to either IC or UC group based on their address of permanent residence. Patient data were registered at discharge and 6, 12, and 24 months after discharge from the TUH.

#### The allocation

The city of Trondheim has 173.000 inhabitants. The municipal primary healthcare is organised in four districts; the biggest district (Lerkendal) was matched with the smallest divistrict (Heimdal) with approximately 83 000 inhabitants, and the two divisions Østbyen and Midtbyen made a pair of approximately 75 000 inhabitants. Østbyen / Midtbyen became the intervention districts by lottery. The demography is quite similar according to age and disease panorama, i.e. the number of inhabitants 55–79 years old are the same in the two district pairs (Lerkendal / Heimdal; 15 800 and Østbyen / Midtbyen 15 200).

#### The intervention

The core elements of the COPD-Home intervention were: (a) a call center for support and communication with the patients and home-care nurses; (b) an education session for the home-care nurses and an interactive e-learning program for the patients; (c) an individualized self-management plan for the patients, and (d) joint visits at the patients`homes by a specialist nurse who repeated the core element of the educational program and reinforced the specific health behaviors as well as making necessary changes in the patients`treatment program (12).

#### Measures

Data on patient characteristics including age, sex, co-morbidity, forced expiratory volume in one second (FEV1), the body mass index (BMI), medical treatment and information about living arrangements and cigarette smoking were collected from the study reports and medical charts.

The St. George’s respiratory questionnaire (SGRQ) is a disease- specific instrument designed to measure QOL in patients with lung disease and consists of 76 items divided into three components: symptoms, activity and impact. Scores for each of the three components are calculated, as well as a total SGRQ score. The scores range from 0 to 100, with higher scores indicating worse QOL [14]. A four-unit change in the total score is considered as a minimal clinically important difference (MCID) for this instrument [15]. Scores from the SGRQ questionnaire are reproducible and sensitive to changes over extended time periods [16].

The Hospital Anxiety and Depression Scale (HADS) is a self–assessment scale that was developed to identify anxiety and depressive moods separately among patients in non-psychiatric clinics [17]. The HADS scale has a total of 14 items, 7 items for the anxiety (HADS-A) subscale and seven items for the depression (HADS-D) subscale. Each item is rated on a 0 (not at all) to 3 (very much) scale. Subscale scores for HADS-A and HADS-D can range from 0 to 21. Higher scores indicate higher levels of anxiety or depression [18, 19]. In order to discriminate cases from both non-cases and borderline cases a cut–off score of ≥ 8 in each subscale is recommended [20]. The proposed minimal important difference (MID) of the HADS–A and HADS-D scores in COPD patients are 1.32 and 1.40 respectively [21].

Patient Activation Measure (PAM) is a tool for measuring the level of patient engagement in their own healthcare and consists of 13 items assessing knowledge, skills and confidence in self-care [22]. PAM is the only validated instrument that comprehensively measures the degree to which patients are activated to manage their own health care. The PAM summary score range from 0 to 100, and patient activation measured by PAM is generally divided into four stages of activation: (1) PAM score ≤ 47.0: people tend to be overwhelmed and unprepared to play an active role, they are predisposed to be passive recipients of care; (2) PAM score 47.1–55.1: individuals lack knowledge and confidence for self-management; (3) PAM score 55.2–67.0: people are beginning to take action but may still lack confidence and skills to support new behaviors; (4) PAM score ≥ 67.1: people have confidence and perform adequate behaviors, but may not be able to maintain them in the face of stress [23]. Commonly, a difference in PAM score of at least 5 points is regarded as adequate to separate healthy from less healthy behaviors [24].

#### Ethical approval

The study was approved by the Regional Ethics Committee (REC), and all participants gave their written informed consent.

#### Statistical analyses

The SGRQ, PAM and HADS questionnaires were scored according to the developer`s guidelines, and the recommended methods for handling of missing items were used. The changes in scores were calculated as the difference between scores at baseline and scores after 6, 12 and 24 months of follow-up. The principal comparisons of changes were performed on an intention-to treat basis. Normality of the continuous variables was tested by the Kolmogorov-Smirnov test. The continuous variables for clinical parameters were expressed as mean ± SD or mean plus 95% confidence interval (95%CI). Independent–samples *t-test* was used to compare the means of some continuous normally distributed variables. The non-parametric equivalent of the independent samples t-test (The Mann-Whitney U test) and paired t-test (The Wilcoxon matched pairs test) were used to compare not normally distributed data from different groups. The Chi-square test and Fishers exact test were used to compare proportions. The Spearmen correlation coefficient (rho) was calculated to determine the strength of the relationship between the baseline PAM score and the independent variables sex, age, smoking status and HADS-D, HADS-A, and scores for each of the three components of SGRQ. Logistic regression was performed to assess the individual impact of the above- mentioned independent variables on the dependent variable—dichotomized PAM score at baseline (0: score ≥ 55.2; 1: score ≤ 55.1).

P-values of less than 0.05 were considered to be statistically significant. Statistical analyses were carried out through the use of computer IBM software SPSS 21 (Chicago, IL, USA).

## Results

### Subjects and baseline characteristics

**Fig 1:**
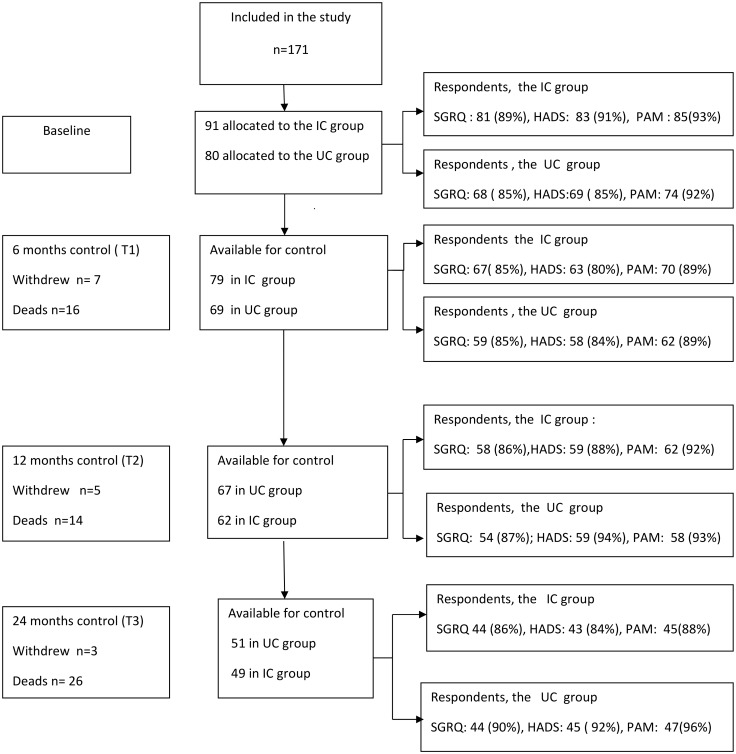
Schematic presentation of participant and number of respondents in percent (%) to participants in the study during 24 months of follow-up. IC: integrated care; UC: Usual care; SGRQ: the St.George’s Respiratory Questionnaire; HADS: the Hospital Anxiety and Depression Scale; PAM: the Patient Activation Measure.

Of the 199 patients who were invited to participate, 27 patients declined, and 171 (86%) patients were randomly allocated to either the IC group (n = 91) or the UC group (n = 80). A flow chart of the number of participants and response rates is shown in Fig 1.

There were no significant differences in the baseline characteristics between the IC or the UC groups with regards to age, gender, BMI, lung function, COPD severity, the number of current cigarette smokers, the number of persons living alone, the burden of comorbidities or the number of participants receiving home nursing (see Table 1).

**Table 1:** Subjects´ and diseases´ characteristics at baseline.

Variables		IC group n = 91	UC group n = 80	P-value
Age, years, Mean (SD)		73.6 (9.2)	72.2 (9.4)	0.38
Sex, n (%)	Male	39 (42.9)	34 (42.5)	0.96
	Female	52 (57.1)	46 (57.5)	
BMI, kg/m^2^, Mean (SD)		22.7 (5.2)	23.5 (6.0)	0.55
FEV1 L/sec, Mean (SD)		0.86 (0.36)	0.84 (0.33)	0.68
FEV1%, Mean (SD)		33.6 (9.9)	33.4 (9.4)	0.92
GOLD3, n (%)		54 (59.3)	46 (58.5)	0.81
GOLD 4, n (%)		37 (40.7)	34 (42.5)	
Hospital admissions (number) year prior study:	1	60 (65.9)	46 (57.5)	0.26
	2–3	23 (25.3)	23 (28.8)	0.61
	≥4	8 (8.8)	11 (13.8)	0.30
Living alone n (%)		47 (51.6)	38 (47.5)	0.58
Current smokers, n (%)		30 (33.0)	32 (40.0)	0.46
Receiving home care, n (%)		51 (56.0)	41 (51.3)	0.53
Charlson co morbidity Index	1–3	18 (19.8)	18 (22.5)	0.87
	4–6	57 (62.6)	47 (58.8)	
	7–10	16 (17.6)	15 (18.8)	
Inhaled medication, n (%)	LAMA	20 (39.2)	25(51)	0.2
	LABA+ICS	36 (70.6)	35 (71.4)	0.9

IC: integrated care; UC: usual care; BMI: body mass index; FEV1: forced expiratory volume in one second; FEV1%: FEV1% of predicted value; HA: hospital admission due to AECOPD one year prior study start; LAMA: long –acting muscarinic receptor antagonist; LABA: Long- acting β2-agonist; ICS: inhaled Corticosteroids.

### Health related QOL, SGRQ

The mean values of SGRQ total scores and symptom scores were statistically different between the IC and the UC group at baseline: 57.3 versus 63.2 (p = 0.01) and 63.0 versus 71.8 (p = 0.002) respectively (see Table 2).

**Table 2:** The mean SGRQ, HADS, PAM scores and difference in score change over time.

		Mean scores (95%CI)								Mean difference in change between IC and UC groups (95%CI)		
Time points		T0		T1		T2		T3		T1	T2	T3
Group		IC	UC	IC	UC	IC	UC	IC	UC			
**SGRQ**	Total score	57.3^a^ (53.8 to 60.9)	63.2^a^ (58.9 to 67.5)	53.6 (49.1 to 57.9)	55.4 (50.0 to 60.8)	56.5 (52.0 to 61.0)	57.6 (52.3 to 63.0)	53.2 (47.5 to 58.9)	60.4 (53.7 to 67.1)	2.3 (-2.6 to 7.2)	4.9 (-1.0 to 11.0)	-1.2 (-7.8 to 5.3)
	Symptoms	63.0^a^ (59.0 to 66.7)	71.8^a^ (67.0 to 76.0)	58.2 (53.0 to 63.4)	60.3 (54.1 to 66.5)	61.2 (55.6 to 66.8)	66.2 (60.8 to 71.7)	55.0 (48.2 to 62.0)	65.8 (59.0 to 73.0)	3.9(-3.4. to 11.3)	2.9(-4.3 to 10.1)	-0.8 (-8.6 to 7.0)
	Activity	76.8 (72.7 to 80.7)	76.1 (70.5 to 81.7)	72.6 (67.8 to 77.5)	71.5 (65.7 to 77.2)	74.6 (69.7 to 79.6)	73.1 (67.2 to 79.0)	72.4 (66.0 to 78.9)	75.6 (68.5 to 82.5)	-0.1 (-6.5 to 6.2)	0.6 (-7.9 to 6.6)	-5.7 (-13.0 to 1.5)
	Impacts	46.4 (42.3 to 50.5)	51.2 (46.1 to 56.3)	41.5 (36.6. to 46.4)	44.9 (39.1 to 50.8)	45.5 (40.5 to 50.6)	47.0 (41.4 to 52.7)	42.2 (35.7 to 48.6)	50.7 (43.6 to 57.8)	1.5 (-3.9 to 6.9)	4.8 (-1.8 to 11.4)	-1.4 (-8.9 to 6.0)
**HADS-A**		6.5 (5.46 to 7.4)	7.2 (6.0 to 8.5)	6.6 (5.5 to 7.6)	6.4 (5.1 to 7.7)	5.7 (4.7 to 6.6)	7.2 (5.8 to 8.7)	5.7 (4.4 to 6.9)	7.1 (5.3 to 8.8)	0.4 (-0.8 to 1.6)	-1.4^b^ (-2.7 to -0.09)	-0.9 (-2.8 to 0.1)
**HADS-D**		6.1 (5.4 to 6.7)	6.0 (5.0 to 7.0)	6.1 (5.2 to 6.9)	6.3 (5.2 to 7.4)	6.0 (5.0 to 7.0)	6.5 (5.5 to 7.6)	5.9 (3.9 to 7.9)	5.0 (3.8 to 6.1)	-0.3 (-1.4 to 0.86)	-1.0^b^ (-2.1 to 0.18)	0.2 (-1.8 to 2.2)
**PAM**		69.2 (66.0 to 72.3)	66.7 (62.7 to 6.7)	72.9 (69.6 to 76.2)	71.3 (67.2 to 75.3)	72.5 (68.7 to 76.2)	70.4 (66.4 to 74.4)	75.0 (70.4 to 79.7)	71.0 (65.9 to 76.2)	0.5 (-6.2 to 7.3)	0.5 (-5.8 to 6.7)	3.1 (-4.5 to 10.8)

T0: baseline; T1: after 6 months of follow-up; T2: after 12 months of follow-up; T 3: after 24 months of follow-up; IC: integrated care; UC: usual care; SGRQ: The St. Georg’s Respiratory Questionnaire; HADS –A: the Hospital Anxiety and Depression Scale, score for Anxiety; HADS –D: the Hospital Anxiety and Depression Scale, score for Depression; PAM: the Patient Activation Measure;

^a^: statistically significant difference in Mean scores between IC and UC groups, p = 0.01 for Total score and p = 0.002 for Symptoms score;

^b^: statistically significant difference in mean score change between IC and UC groups, p = 0.02 for HADS-A and p = 0.03 for HADS-D.

During the first 12 months of follow-up the mean values of the total scores became more levelled between the IC and the UC group, mainly due to a reduction of the symptom and impact scores in the UC group. While the activity, impact and total scores continued to be lower compared with the baseline values in the IC group, in the UC group there was a trend towards an increase in these scores during the last 12 months of follow-up. The regarded clinically significant four–unit change in the total score was achieved only in the UC group: mean change -5.2 (95%CI -9.0 to-1.4) after 6 months and mean change -4.2 (95%CI -8.3 to 0.07) after 12 months. There were no statistically significant differences in the mean change in either the component or total scores between the IC and the UC group during follow-up.

### Anxiety and depression, HADS-A and HADS-D

There were no significant differences between the IC and the UC group in the mean HADS-A or HADS-D scores at baseline or during follow-up (see Table 2). However, after 12 months of follow-up there was a trend towards a reduction in the mean HADS–A score in the IC group compared to the UC group, resulting in a significant difference in the mean change: -0.9 (-1.8, - 0.01) in the IC group versus 0.5 (-0.4 to 1.4) in the UC group, p = 0.02. Further, after 12 months of follow-up the HADS-D scores remained stable in the IC group, but tended to increase in the UC group, resulting in a significant difference in the mean change in scores during this period: 0.05 (-0.8,0.9) in IC group versus 1.0 (0.3 to 1.8) in UC group, p = 0.03. The MID of the HADS –A and HADS-D score compared with baseline was not achieved either in the IC or the UC group.

### Measure of patient activation, PAM

There were no significant differences between the IC and the UC group in the mean values of the PAM scores neither at baseline nor during follow-up (see Table 2). After 24 months of follow-up there was a tendency towards an increase in the scores in both groups, but the mean change in scores reached clinical significance only in the IC group; 6.7 points (95% CI 0.7 to 12.6) compared to 3.6 (95% CI -1.4 to 8.6) in the UC group. There were no significant differences between the IC and the UC group in the distribution of patients between the activation stages 1–4 neither at enrolment nor during follow-up.

### The association between PAM score, hospital admissions, HADS-A, HADS-D, and SGRQ scores

There was no significant association between the PAM scores at baseline and the number of hospital admissions during follow-up. Correlation analysis of baseline scores showed a negative association between both PAM and HADS-A and HADS-D scores (n = 159, rho = -0.202, p = 0.01 for HADS –A and n = 159, rho = -0.389, p = 0.01 for HADS –D) and between PAM scores and SGRQ impact component scores (n = 155, rho = -0.328, p = 0.01). Moreover, there was a negative association between PAM scores and current cigarette smoking (n = 159, rho = -0.17, p = 0.03). In a logistic regression model including sex, age, HADS-A, HADS-D and SGRQ impact component score, the strongest predictor of reporting a lower PAM score (≤ 55.1) was the HADS–D score and current cigarette smoking (see Table 3). Higher HADS-D score and current cigarette smoking significantly increased the odds for a low PAM score.

**Table 3:** Logistic regression analysis of lower patient activation measure (PAM) score.

N = 148				
Predictors	Odds Ratio	95% Confidence interval for Odds Ratio		p-Value
		Lower	Upper	
**Age**	1.02	0.96	1.07	0.48
**Male**	0.45	0.16	1.19	0.10
**Current smoker**	3.07	1.15	8.15	0.02
**HADS -A**	1.05	0.92	1.21	0.40
**HADS- D**	1.22	1.03	1.45	0.02
**Impact**	1.00	0.99	1.00	0.27

HADS-A: the Hospital Anxiety and Depression Scale, score for Anxiety; HADS—D: the Hospital Anxiety and Depression Scale, score for Depression; Impact: Impact component of the St.George`s respiratory questionnaire

## Discussion

To the best of our knowledge this is the first study where patient related outcomes of an IDM intervention program has been evaluated using the patient activation measure (PAM) together with the acknowledged SGRQ and HADS questionnaires. Health related QOL measured by SGRQ, was significantly better at enrolment and relatively more stable throughout the follow-up period in the IC group compared to the UC group. However, no significant differences in the changes in the SGRQ scores occurred between the groups during follow-up.

In recent years the impact of IDM programs on health related QOL measured by the SGRQ has been evaluated in several studies. Chavannes et al [25] showed that IDM improved QOL after one year compared to usual care among patients with COPD in primary care. The improvement in SGRQ was both clinically relevant and statistically significant, and was greatest in patients with dyspnea at small efforts, expressed by an MRC score > 2. Possibly, the room for improvement in QOL was more diminished among the participants in the present study who were older and had more severe COPD compared to the above study. The findings by Rice et al [26] are more comparable to the present study with respect to age and COPD severity. In keeping with the results from the present study they showed that after one year health related QOL measured by SGRQ remained relatively stable in the disease management group, but in contrast to the present study, the SGRQ score increased by an average of 6.4 points in the usual care group. More recently, Kruis et al [7] investigated the long term effectiveness of an IDM intervention on health status measured by the Clinical COPD Questionnaire (CCQ), SGRQ and daily physical activity measured by International Physical Activity Questionnaire (IPAQ) in primary care. The participants had significantly lower total SGRQ scores at baseline compared to the participants in the present study, which may be explained by the fact that only 22% of the patients belonged to COPD GOLD stage III or IV. However, in keeping with our findings their results showed no improvement in QOL measured by either CCQ or SGRQ, but at 12 months the proportion of patients with moderate or high activity levels measured with the IPAQ had improved significantly in the intervention group compared with the controls. In contrast to SGRQ where the degree of impairment of daily activities is scored, the IPAQ measures changes in performed physical activities. Thus, IPAQ may be a more sensible instrument to assess the results of self-management programs among patients with severe limitations in lung function and physical condition. One explanation may be that while self-management programs are designed to empower the participants and enable them to improve their management of a chronic disease [27] the measured outcomes are often so called distal desired outcomes such as symptoms, and physical, psychological and social function measured by ex. SGRQ or CCQ [14, 28]. On the other hand IPAQ and PAM are designed to evaluate more proximal outcomes, i.e. in what degree the patients actually perform healthy activities. Thus, in future research maybe primary outcomes should be changed towards measurements that capture the actual change in the patients’ activities or knowledge or confidence to perform healthy activities such as IPAQ or PAM. Moreover, one may hypothesize that it is unrealistic to expect clinically significant improvements in health related QOL in old patients with severe disease, irrespective of the intervention. Prevention of a reduction in QOL over time may be regarded as a success.

Although there was no reduction in hospital admissions in the UC group in the present study, there was an improvement in the SGRQ total score between six and 12 months of follow-up. This could partly be explained by the fact that they were hospitalized due to an AECOPD at inclusion which is known to temporarily worsen QOL[29, 30], and partly by being enrolled in a clinical study which implies being favored with more attention from the study staff during follow-up [31].

In the present study a temporary positive effect of the intervention on the levels of both anxiety and depression was shown. These findings are consistent with the results from the trial by Bucknall et al of self-management among patients with moderate to severe COPD [32] although the mean baseline HADS-A (9.3–10.0) and HADS-D (8.3–8.5) scores were higher in their study compared to the corresponding scores in the present study (6.5–7.25 versus 6.0–6.1). Interestingly, in the study of self-management in patients with COPD Schüz et al [33] found that knowledge mediated the effect of the intervention on changes in physical activity only among participants reporting low levels of anxiety or depression. The authors suggest that patients with elevated anxiety or depression may need to be treated appropriately before engaging in chronic disease self-management interventions. In according with the above findings we may speculate that the relatively low level of symptoms of anxiety and depression among the participants in the present study may have contributed to a sound basis for the implementation of the COPD-Home intervention.

In the present study the degree to which the patients were engaged in managing their own health care was measured using the PAM. The mean PAM scores at baseline were comparable to the mean PAM scores of an American group (61.9), a Danish group (64.2), and a Dutch group (61.3) of people with diagnosed chronic diseases or moderate to severe levels of physical disability [34], but was higher than the PAM scores of the Dutch COPD patients from the most recent study of Bos-Touwen et al [35]. In the present study there was an increase in the PAM scores from baseline in both the IC and the UC group, but the achieved change in score was clinically significant only in the IC group.

Although no association between PAM score and the number of hospital admissions was found, the clinical significant increase in PAM score in the IC group during follow-up coincided with the 46.5% reduction in the number of hospital admissions during follow-up in the same group [13]. Hence, we may speculate that the COPD-Home intervention contributed to the individuals in the intervention group by enhancing their understanding, skills and confidence in managing their own health.

Our results failed to show a significant association between PAM scores and SGRQ scores which may imply that the level of health related QOL do not influence the degree of patients`engagement in their own health care.

Unlike other studies (25, 34) we were able to show that current smoking significantly increases the odds of a low PAM score. The finding of a negative association between the HADS-D and the PAM scores is in keeping with previous studies showing that patient activation was related to both physical and mental health components [24], and that increased patient activation was associated with a decrease in the severity of reported depressive symptoms [36, 37]. Hence, it may be reasonable to consider screening of the participants`depressive symptoms before enrollment into IDM programs.

### Limitations of the study

Some limitations of the study ought to be considered. First, the study population is rather small and was restricted to a relatively high-risk group of patients with COPD, and thus it is uncertain to what extent the COPD-Home intervention would be effective in patients with milder disease. Secondly, the total SGRQ score and the symptoms sub score were statistically significantly lower in the IC group at baseline compared to the UC group indicating that the patients in the IC group had a better functional capacity. Such bias may indicate a systematic selection bias. Due to the inclusion and allocation procedures in the present study the participants were randomized according to their address of residence. The municipality of Trondheim is organized into four districts, and the city is known to have few socioeconomic demarcations between the districts. The two district pairs were chosen to level out differences in the disease and age panorama. Hence, the authors conclude that selection bias did not occur and that the higher SGRQ score in the IC group is an accidental finding.

### Strengths of the study

The strength of the present study is the clinical validity of the study population. The participants represent a real life clinical COPD population due to very few exclusion criteria and a high response rate. Secondly, the study adds to the previous very scarce knowledge of patient activation measured by PAM score among COPD patients and the association between PAM score and SGRQ- and HADS scores in this patient group.

## Conclusions

The COPD–Home IDM intervention did not result in any statistically significant changes in mean SGRQ, HADS-A, HADS- D or PAM scores during the 24 months of follow-up.

## Supporting Information

S1 Text (PDF)

S2 Text (PDF)

S3 Text (DOC)

S4 Text (DOCX)

S1 Table (PDF)

S2 Table (XLSX)

## Abbreviations

AECOPD: acute exacerbation of COPD
BMI: Body Mass Index
COPD: chronic obstructive pulmonary disease
DTM: Department of Thoracic Medicine
FEV_1_: forced expiratory volume in one second
FEV_1_%: FEV_1_ percent of predicted value
GP: general practitioner
HA: hospital admission
HADS: Hospital Anxiety and Depression Scale
HD: in-hospital days
IC: integrated care
IQR: inter-quartile ranges
MID: minimal important difference
PAM: Patient Activation Measure
QOL: quality of life
SGRQ: St. George’s Respiratory Questionnaire
TUH: Trondheim University Hospital
UC: usual care
