# Supplementary material for: Does an Integrated Care Intervention for COPD Patients Have Long-Term Effects on Quality of Life and Patient Activation? A Prospective, Open, Controlled Single-Center Intervention Study
Source: PLoS One. 2017 Jan 6;12(1):e0167887. doi: 10.1371/journal.pone.0167887 (PMC5218408; doi:10.1371/journal.pone.0167887)
Supplement: S1 Text — (PDF) [file pone.0167887.s001.pdf]

Scandinavian Journal of
Caring Sciences
EMPIRICAL STUDIES
doi: 10.1111/scs.12069

The development of an integrated care model for patients with severe or very severe chronic obstructive pulmonary disease (COPD): the COPD–Home model

Synnøve Sunde RN[1], **Rolf Aksel Walstad** MD, PhD[1,2], **Signe Berit Bentsen** RN, PhD[3], **Solfrid J. Lunde** RN[1], **Eva Marie Wangen** RN[1], **Tone Rustøen** RN, PhD[4,5] and **Anne Hildur Henriksen** MD, PhD[1,2]

[1]Department of Thoracic and Occupational Medicine, Trondheim University Hospital, Trondheim, Norway, [2]Institute of Circulation and Medical Imaging, Norwegian University of Science and Technology (NTNU), Trondheim, Norway, [3]Department of Research, Haugesund Hospital, Helse Fonna, Haugesund, Norway, [4]Department of Research and Development, Division of Emergencies and Critical Care, Oslo University Hospital, Oslo, Norway and [5]Department of Nursing Science, University of Oslo, Oslo, Norway

Scand J Caring Sci; 2014; 28; 469–477

The development of an integrated care model for patients with severe or very severe chronic obstructive pulmonary disease (COPD): the COPD–Home model

Background: Adherence to guidelines for managing stable chronic obstructive pulmonary disease (COPD) and its exacerbations is inadequate among healthcare workers and patients. An appropriate care model would meet patient needs, enhance their coping with COPD and improve their quality of life (QOL).
Aim: This study aims to present the 'COPD–Home' as an integrated care model for patients with severe or very severe COPD.
Model: One principle of the COPD–Home model is that hospital treatment should lead to follow up in the patient's home. The model also includes education, improved coordination of levels of care, improved accessibility and a management plan. One of the main elements of the COPD–Home model is the clear role of the home-care nurse. Model development is based on earlier research and clinical experience. It comprises: (i) education provided through an education programme for patients and involved nurses, (ii) joint visits and telephone checks, (iii) a call centre for support and communication with a general practitioner and (iv) an individualised self-management plan including home monitoring and a plan for pharmacological and nonpharmacological interventions.
Conclusion: The COPD–Home model attempts to cultivate competences and behaviours of patients and community nurses that better accord with guidelines for interventions. The next step in its development will be to evaluate its ability to assist both healthcare workers and planners to improve the management of COPD, reduce exacerbations and improve QOL and coping among patients with COPD.

Keywords: chronic obstructive pulmonary disease (COPD), integrated care, academic detailing, self-management, systematic prevention strategies, chronic obstructive pulmonary disease, outreach nursing.

Submitted 22 January 2013, Accepted 16 July 2013

Introduction

Chronic obstructive pulmonary disease (COPD) is a lung disease characterised by chronic obstruction of lung airflow that interferes with normal breathing (1). As lung function worsens, exacerbations tend to occur with increasing frequency (2), often requiring hospitalisation for advanced treatment. The severity of COPD is mainly defined by lung function measured by spirometry where the forced expired volume during 1-second (FEV1) is calculated according to a predicted value for each patient. The Global Initiative for Chronic Obstructive Lung Disease (GOLD) criterion for COPD classifies the disease in stages I–IV (mild, moderate, severe and very severe) according to the value of FEV1 as per cent of predicted value (1). Frequent exacerbations lead to increased mortality, with a 5-year survival rate of 40–50% (3). In 1995, the number of hospitalisations due to exacerbation

Correspondence to:
Anne Hildur Henriksen, Department of Thoracic and Occupational Medicine, Trondheim University Hospital, Olav Kyrres gt. 17, 7006 Trondheim, Norway
E-mail: anne.hildur.henriksen@stolav.no

© 2013 Nordic College of Caring Science

of COPD in Norway was more than 15 000, of which 53% were over 65 years old (4). For frail elderly patients, hospitalisation carries risks (5) of functional decline or other complications (6). Also, a decline in quality of life (QOL) has been identified in those with severe to very severe COPD (7) and particularly following acute exacerbation (2). In addition, an increased prevalence of depression and anxiety postdischarge has been found (8). To reduce the number of hospital admissions and improve patient QOL, self-management programmes should better meet patient needs.

Previous research has revealed that the organisation of primary care and nurse staffing is significantly associated with improved clinical care of patients with COPD (9, 10). Moreover, Ketelaars et al. (11) show that specialised community nurses demonstrate a higher clinical competence in the care of patients with COPD than do generalist nurses. In Norway, local authorities provide free home-care nursing programmes. Both authorised nurses and other healthcare workers (home-care nurses) are employed by municipalities to visit persons with various needs in their own home. Home-care nurses who administer medication, provide meals or assist in bathing visit many persons with severe COPD. The authors hypothesise that when equipped with adequate knowledge, tools and communication for backup support to effectively respond to patients' needs, home-care nurses can play an important role in home monitoring of symptoms associated with COPD exacerbations. Their work may result in reducing the number of COPD exacerbations per year, reduction in the overall use of healthcare services, increased QOL, less anxiety and depression, and improved coping. The World Health Organization defines 'integrated care' as a concept bringing together inputs, delivery, management and organisation of services related to diagnosis, treatment, care, rehabilitation and health promotion. Integration is a means for improving services in relation to access, quality, user satisfaction and efficiency (12).

Based on the above considerations, the aim of this study is to develop, implement and evaluate the COPD–Home model as an integrated means of bringing patients' and primary healthcare workers' competences and behaviours more in line with guidelines for pharmacological and nonpharmacological interventions. In this study, the COPD–Home model will be described. Study results will be presented in a following paper.

Background

Healthcare workers are challenged to identify a care programme that can modify the specific features of the patient's total situation in order to improve their adherence to pharmacological and nonpharmacological intervention recommendations.

Design and randomisation procedure

Several randomised controlled trials (RCT) aiming to reduce hospitalisations and to improve QOL have been conducted (13–15). While Bourbeau et al. (14) randomised patients into usual care or self-management, Koff and co-authors (13), Casas et al. (15) and Garcia-Aymerich (16) randomised patients into usual care or integrated care. In the study by Skwarska (17), patients were randomised to home support or admitted to hospital in the standard manner, while Shepperd (18) randomised patients to hospital-at-home care or inpatient hospital care.

Duration of follow-up

The duration of follow-up in the integrated care/self-management studies was most often 12 months (14–16), whereas one study lasted for only 3 months (13). While in the study by Skwarska (17), patients receiving treatment and care at home were assessed 8 weeks after the initial assessment, and the final follow-up in Sheppard's study of hospital at home was at 3 months (19).

Intervention and findings

Previous studies have shown that patients with COPD benefit from regular follow-up and education (13, 14, 20, 21). Koff and co-workers (13) showed how disease-specific education, teaching self-management techniques, enhanced communication and remote home monitoring improved QOL and reduced healthcare costs in a 3-month period. At enrolment, patients with COPD stage 3 or 4 were given the education by the coordinator and reinforced daily by telephone. Participants spent 20 minutes every morning on the education programme and recording observations. Participants' daily lung function observations were transferred to the coordinator.

Bourbeau (14) gave a COPD-specific self-management programme to the intervention group in their home 1 hour per week for 8 weeks. The case manager made weekly telephone calls for the first 8 weeks and then monthly calls over the 12-month study period. The programme also included an exercise programme that should be followed at least three times a week and an action plan for acute exacerbations. The case manager was available to the intervention group by telephone. This intervention significantly reduced the utilisation of healthcare services, improved health status and reduced COPD-related hospital admissions at 12-month follow-up by 40% in the intervention group compared with the normal-care group (14).

The beneficial effects of this approach have been confirmed in patients with COPD in Belgium and Spain (15, 16). In these studies, the interventions included

© 2013 Nordic College of Caring Science

education, coordination of levels of care, improved accessibility after discharge supported by a customised care plan shared with the primary care team and access to a specialised case manager nurse through a web-based call centre. At 1-year follow-up, Garcia-Aymerich et al. (16) concluded that this integrated care trial improved patients' understanding of the disease and treatment adherence, suggesting that these factors may play a role in the prevention of severe COPD exacerbations that trigger hospitalisation. In a study by Casas et al. (15), the rate of hospitalisation was lower in the intervention group than in the control group, and the number of patients not requiring readmission was higher in the intervention group than in the control group (49 vs. 31%).

Skwarska et al. (17) show that home-supported discharge is a well-tolerated, safe and economic alternative to hospital admission for a proportion of patients with exacerbation of COPD. Nissen and Jensen (22) came to a similar conclusion in their study of nurse-supported discharge of 108 patients with COPD exacerbations.

One well-described model of integrated care is the 'hospital-at-home' service (18, 19) that provides healthcare professionals for limited active treatment in the patient's own home for a condition that normally would require acute in-hospital care (19). This model offers a full substitute for acute hospital care to patients with COPD who are assessed for their appropriateness for hospital at home in the emergency department or ambulatory site. Diagnostic procedures, such as X-ray, and treatments, such as oxygen therapy and administration of intravenous fluids in the patient's home, are offered by the hospital-at-home team. The hospital-at-home physician makes one or more home visits per day. Together with the hospital-at-home nurses, they are also available 24 hours/7 days a week to attend emergencies until discharge from the 'hospital-at-home' service (23).

'Early discharge hospital at home' is another service providing active treatment by healthcare professionals in the home following discharge for patients that otherwise would have remained in an acute hospital ward (18). These services are usually provided by a hospital-at-home team of healthcare professionals, offering the same care as the hospital at home, but following initial treatment in a hospital unit.

Disease severity

The severity of COPD among the subjects varied from moderate to very severe. Some examples are Koff 's (13) criteria on FEV1 for inclusion was a value up to 50% of the expected value (COPD stages 3–4), and Bourbeau's (14) criteria on FEV1 was patients with a FEV1 ranging from 25 to 75%. The baseline FEV1 in the study by Casas was 43% of predicted in the integrated care group and 41% of predicted in the usual care group (15). The mean FEV1 of patients randomised to home support in Skwarska's study was 0.77 l, and for those admitted to in-hospital treatment, mean FEV1 was 0.66 l (17).

Differences in research design create challenges for comparing the findings of studies in this area. The severity of COPD in participants, the elements of the interventions and their different emphases, as well as the length of follow-up all vary across studies. A recent Cochrane review on outreach nursing for patients with COPD showed that home care improved QOL, but had an unpredictable effect on the risk of being admitted to the hospital (24). The review included nine studies. They were all based on an outreach nurse visiting the patient in their home, providing support, education and monitoring health. The length of the studies ranged from 3 to 12 months. The COPD severity differed between the studies. The review concludes that more long-term research is needed to confirm the usefulness of home visits by a respiratory healthcare worker for patients with COPD (24).

Finally, earlier research indicates that education, regular follow-up and reinforcement, easy access to healthcare providers and home monitoring with individual self-management plans could be cornerstones of an integrated care model for patients with severe or very severe COPD. Based on former scientific studies and clinical experience, the COPD model was developed.

The COPD–Home model

Theoretical framework

The GOLD report, Global Strategy for the Diagnosis, Management, and Prevention of COPD (1), is based on the most highly validated current concept of COPD pathogenesis and the available evidence on the most appropriate management and prevention strategies. It concludes that there is considerable evidence to show that management of COPD is generally not carried out in accordance with current guidelines. According to the GOLD report, an effective COPD management plan should include assessing and monitoring the disease, reducing risk factors, managing stable COPD and managing exacerbations (1). The guidelines suggest that integrated care interventions that include education, coordinate levels of care and improved accessibility have shown positive effects on QOL and hospital admissions.

During the trajectory of COPD, patients are constantly learning the new behavioural skills required for appropriate self-management (25, 26). The patient's understanding of the disease and their involvement in its management and self-care on an ongoing basis are the key features of self-management. In COPD, disease-specific self-management skills are essential in order to monitor symptoms and detect the onset of repeated and increasingly severe acute exacerbations (1, 26–28).

© 2013 Nordic College of Caring Science

Detection and recognition of worsening symptoms, such as breathlessness, increased and more purulent sputum and cough, also form the initial stage of implementing an action plan to treat an exacerbation (1, 28).

Soumarai and Avorn (29) define the theory and practice in developing methods to improve physicians' clinical decision-making as 'academic detailing'. The educational programme in COPD–Home is based on such techniques. The following approaches are included in this technique: (i) investigating baseline knowledge and motivations for current behaviours, (ii) defining clear educational and behavioural objectives, (iii) establishing credibility via a respected individual who delivers the educational material, (iv) stimulating the active participation of the learner during educational interactions, (v) using concise theoretical materials, (vi) highlighting and repeating the essential messages and (vii) providing positive reinforcement of behavioural changes in follow-up visits. In the COPD–Home education programme, the academic detailing techniques are combined with the pedagogic strategy of 'outreach education', defined as one or more personal visits by a trained person to a healthcare provider in the provider's own setting (29). Nurse coaching carried out in COPD–Home is appropriate for these techniques and strategies.

Study design

COPD–Home is a prospective, open, clinical intervention study designed to measure the effect of integrated care on increasing patients' and community nurses' deliberations and behaviours in accordance with current guidelines for COPD management. Patients with severe and very severe COPD (Gold III–IV) that had been hospitalised due to COPD exacerbation in the Department of Thoracic Medicine (DTM) or in the observation unit were invited to participate in the study. The inclusion criteria were that the subjects must be able to cooperate, be able to sign an informed consent form and not suffer from another severe disease with an expected lifespan of less than 6 months. Moreover, those receiving care from a home-care nurse were required to have scheduled a minimum of one visit per week.

Eligible patients were randomised into an intervention group and a control group before discharge from the hospital. To address variation in patients receiving home-care nursing, we divided the intervention group into two categories: intervention 1 for those who received care from a community nurse, and intervention 2 for those who did not receive care from a community nurse. In intervention 1, there is closer collaboration between the hospital and the primary healthcare provider, while in intervention 2, there is closer collaboration than usual between the hospital and the patient. In both groups, the general practitioners (GP) received information concerning the interventions, and they were invited to participate in the follow-up. The patients in the control group were also divided into two groups based on whether or not they received home-care nursing. Patients from both groups were followed up according to usual practice by all healthcare providers. The number of specialist nurses from the participating hospital was three throughout the study period, while the number of home-care nurses varied according to the number of participating patients in their city area at the time. The unique protocol ID for the study in the clinical trials registration system is 17417.

Usual care

The standard procedures at discharge from the hospital for the patients with COPD included sending a discharge summary to the GP and the home-care nurse. If suitable and willing to, patients could also be referred to rehabilitation, smoking cessation or training programmes. After discharge from the hospital, patients were requested to return to the outpatient unit in the hospital or attend their GP for a medical examination. The home-care nurse followed up according to decisions made by the primary healthcare provider about the patient's needs. Some patients do not require professional home care, some receive the same level as before hospitalisation and others require additional care.

The elements of the COPD–Home model

In 2008, participants from the University Hospital and the municipality of Trondheim developed the COPD–Home model for integrated care for patients with severe and very severe COPD. The COPD–Home model builds on the GOLD report recommendations for pharmacological and nonpharmacological interventions. One of its principles is that treatment initiated at the hospital does not end on discharge. Instead, following a strengthened follow-up at discharge, treatment is delivered to the patient in their own home for the remainder of their life. The guidelines also suggest a model based on integrated care including: (i) education, (ii) improved coordination of levels of care, (iii) improved accessibility and (iv) a management plan. COPD–Home is based on carrying out these recommendations, with necessary adjustments concerning healthcare organisation in Norway, resources of the project and differences between those who receive care from a home-care nurse and those who do not. One of the main elements of the COPD–Home model is the clear role of the home-care nurse.

The COPD–Home intervention is intended to support the patient and/or the home-care nurse in meeting their needs for monitoring and reinforcement of self-management strategies in stable phases as well as during exacerbations of COPD. To minimise patient stress and their use of healthcare services, these interventions should be

© 2013 Nordic College of Caring Science

carried out at the lowest efficient level. As each person with COPD has distinctive requirements that may change over time, the model must be flexible.

Core elements of the COPD–Home model

Education provided by an education programme for patients and nurses. Home-care nurses are invited as observers to the DTM, over 2 days. They also attend a 3-hour training programme that covers the following:

- Information about the COPD, including pharmacological and nonpharmacological interventions, nursing care and follow-up.
- Training in using an observation form, a plan for pharmacological and nonpharmacological interventions, and PiKo-1 (Electronic Peak Flow metre) for monitoring forced expiratory volume (FEV1).

The patients attend a 15-minute interactive e-learning programme on COPD, which advises on the most appropriate self-management strategy to achieve a stable COPD condition and how to prevent/manage exacerbations. In addition, patients are offered the following:

- Consultation with a specialist nurse regarding individual reactions and questions following the e-learning programme.
- Advice on how to observe personal lung function and register the observations on the observation form.
- Advice on how and when to use the COPD call centre for decision support on interventions or other questions.

Joint visits in the patient's home. One way to increase coordination of levels of care in the COPD–Home model is through joint visits to the patient by the specialist nurse and the home-care nurse. These visits are carried out at approximately three and 14 days post-discharge, and followed up at 6 months, and 1, 2 and 3 years.

When visiting the patient at home, the specialist nurse is responsible for the below mentioned:

- Transfer of knowledge about the pharmacological intervention, how to detect the start of an exacerbation and how and where to call for support.
- Transfer of knowledge of useful, available nonpharmacological interventions and encouraging, coaching and supporting the patient in its application.

The follow-up visits also include examination, making necessary changes in plans for medical and nonmedical interventions and reinforcing desired behaviours.

The patient's GP is invited to participate in visits to the patient's home. When a patient is included in the study, their GP receives a letter about the patient being recruited to the COPD–Home programme, stressing that there is no change in the responsibility for patient follow-up. After discharge from the hospital, the general responsibility for following up the patient remains with the GP. The home-care nurse informs the GP about medical changes made according to the individual plan for pharmacological intervention. Also, when the specialised nurse supervised by a pulmonologist at the DTM acts on symptoms or conditions concerning lung function, the GP is informed by letter from the pulmonologist.

Call centre for support and communication between the levels of care. The call centre is located at the DTM and operated by a specialist nurse who has an open telephone line to a chest physician. The home-care nurse or the patient can call for medical intervention backup or to discuss their condition.

The specialist nurse telephones the patient at least once a month over the 3-year study period and more frequently when the patient has had an exacerbation. These telephone calls intend to reinforce self-management by asking about the patients following up of the medical and nonmedical intervention plan. Notes are taken from these phone calls for follow-up and documentation. The conversations are based on patients needs and do not follow a systematic guide.

Individualised self-management plan. The individualised self-management plan is given to the patient in the first home visit following discharge from the hospital. The plan includes tools to monitor and assess the disease, manage stable COPD, and prevent and manage exacerbations.

1. Monitoring and assessing the disease: The observation form can be used by the patient or home-care nurse to record regular observations on lung function (FEV1 on the device PiKo 1) and other parameters such as physical and mental state, amount and colour of sputum, level of breathlessness, heart and respiratory rate, and temperature.
2. Managing stable COPD and preventing exacerbation: The self-management plan also includes options for nonpharmacological interventions such as the potential for institutional rehabilitation, antismoking programmes, training groups and education, and self-management courses. Nutrition, vaccination and the need for other aids are also suggested together with actions for improvement. Together with the patient, the specialist nurse makes a personal plan for nonmedical interventions and provides the necessary support throughout follow-up. If the patient has a home-care nurse, the nurse will also participate in making these plans for the patient and doing follow-up.
3. Managing exacerbations: Patients also have an individual plan for pharmacological intervention. Both the form and the plan are made using the 'traffic-light system', with normal medication used when the condition is green, stepping up the medication when the condition is yellow and stepping up further when red. The medication needed to follow up the

© 2013 Nordic College of Caring Science

treatment plan is prescribed on discharge from the hospital and is available in the patient's home. Patients are trained to record their clinical observations on a daily basis. If they are unable to record observations themselves, if available, a home-care nurse will record observations on their behalf. Finally, the self-management plan includes useful telephone numbers and a leaflet about living with COPD for the patient and their family.

Discussion

This study is one of the first describing a holistic care approach to patients with severe COPD that includes a clearly defined and independent role for the home-care nurse. In implementing this model, both the specialised nurse and the home-care nurse employ several skills, such as observing symptoms and needs, initiating medical and nonmedical treatments, teaching, coaching and constantly evaluating the patient's condition. Coaching is described as being effective for encouraging, inspiring and empowering patients to reach their maximum health potential (30). The nurses also collaborate with other healthcare professionals depending on the needs and functional level of the patient. Earlier research has revealed that nurses in primary health care sometimes feel insecure in educating, for example COPD patients, miss the support of co-workers and often feel alone with extensive responsibility (31). The COPD–Home model is supposed to increase the level of support of co-workers and the commitment of other professionals as the GP.

The fundamentals of integrated care include adopting a holistic approach to the patient, promoting their self-management, providing an effective response to their needs and sharing of accessible information (32). Terms and concepts such as 'discharge support', 'early discharge' and 'hospital at home' describe different approaches to meeting these challenges (18). Further, there is no consensus on the definitions of these terms and concepts, and they may have different meanings in different countries (33). Many discharge-support schedules tend to be short term, are usually carried out by nurses, intend to reinforce education and encourage compliance with therapeutic measures. The strength of the model presented in this paper is the continuous collaboration and dialogue of the specialised nurse and the home-care nurse with the patient.

In contrast to the two models 'hospital at home' (19) and 'Early discharge hospital at home' (18) where the patients are treated by the 'hospital-at-home team' until stable, patients in the 'COPD–Home' intervention groups are not discharged from the hospital before acute treatment is completed. At discharge from the hospital and during the first period after discharge, patients and nurses are given extra support by the specialised nurse and the chest physician if needed. This is supposed to be an important strength with the COPD–Home model. The home-care nurse gradually assumes responsibility for monitoring and activating treatment of the patient, with only decision support and reinforcement from the call centre. In intervention group 2 (patients without contact with a home-care nurse), the patient and family gradually assume this responsibility, again with decision support and reinforcement from the call centre. Continuous reinforcement is a major element of the model and involves healthcare personnel maintaining regular contact with the patient and their family, observing changes in the situation and acting accordingly.

The patient, family and nurse all need to possess sufficient knowledge to manage medical and nonmedical interventions during stable COPD and following exacerbations. Tools for monitoring symptoms, followed by a plan for easily available medicines and backup support, should be established, and cooperation between healthcare professionals and different levels of care needs to be improved. Home-care nurses are generalists, and their education programme in COPD–Home is limited. As Ketelaars et al. (11) show, a lack of education represents a barrier to a shared care approach. In order to ensure that adequately trained home-care nurses visit the patient with COPD, they are required to possess a minimum of skills. Such skills can be provided in a session on managing COPD in the introduction programme, followed up by annual recertification.

Joint visits to the patient's home represent a challenge to the workload of the GP as home visits are often requested at short notice. On the other hand, if the model is successful, educated patients may be able to solve their problems independently and do not have to see their GP that often for their COPD. One example is the call centre that offers easy access for any requests or for decision support. This is essential for patients having a bad day who may not consider it worthwhile to visit the GP to ask a minor question.

Previous research has shown that family caregivers of patients with COPD provide necessary, cost-effective care for those living with COPD (34). However, they often experience substantial vulnerability in terms of an imbalance between burden and coping capacity (34). When the nurse visits the patients with COPD in their home, they often also meet the family caregiver and can more easily support the family caregiver's needs and efforts.

One limitation of the COPD–Home model is that the call centre is operated by a specialist nurse in the hospital who is active only during weekday business hours. In the event of any need for healthcare services outside these hours, the patient/family/home-care nurse must follow the usual course of contacting the local doctor on call or visiting the emergency unit in the hospital. Our model also depends on the patient/home-care nurse communicating any worsening of symptoms and acting adequately with or without

© 2013 Nordic College of Caring Science

backup support from the call centre. We assume that the physical location of the call centre is of less importance than the accessibility and the skills of the nurse operating the centre. Moreover, the nurse needs easy access to a doctor with sufficient skills regarding COPD treatment. With the aim of obtaining optimum compliance in severely ill patients with COPD, the number of observations required should be confined to the most important symptoms. When needed, the call centre may request more information to fill out the clinical picture. Finally, an electronic version of 'My COPD book' would increase the quality of the nurse coaching through an electronic communication system involving the GP and the hospital. As severe COPD is a lifelong disease, it is essential to create an action plan that can be carried out over an unlimited period of time. The COPD–Home model has the potential for being such a lifelong intervention.

Its major strengths are continuous reinforcement and responsibility for monitoring, followed by adequate action by the patient/family/home-care nurses. The challenge for the home-care nurse is how to organise the working day to maintain continuity for patients. Economic limitations and work-hour regulations often lead to discontinuity in contact between the home-care nurse and the patient. The authors hypothesise that the costs associated with the COPD–Home intervention will be lower than costs generated during a hospital stay or costs associated with the hospital-at-home model.

Clinical observations are recorded in My COPD book. These records are kept on paper and cannot easily be shared between the patient and healthcare workers involved. Nor is an automatic message sent to the call centre if the patient or the community nurse does not write up their daily observations. These aspects represent limitations of the model as currently implemented that contrast with other models (13, 15) that have incorporated a technology platform for the transmission of monitored symptoms.

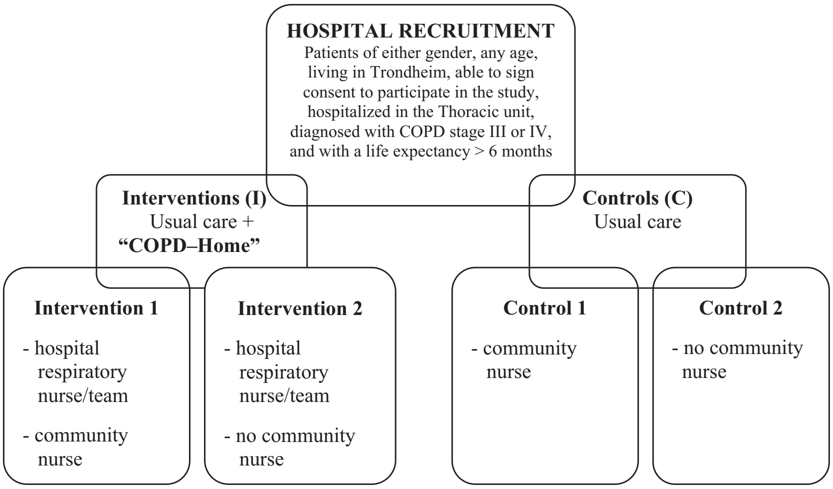

Figure 1 Study design.

COPD – Home<br>Offered in addition to usual care	Usual care in Trondheim, Norway
**Increased degree of integrated care**	**Small degree of integrated care**
a) **education** provided by an education programme for patients and nurses<br>- 15-min interactive COPD education for patients<br>- systematic education of home-care nurses	- when criteria filled; patient offered rehabilitation that includes education<br>- no systematic education of home-care nurses
b) **joint visits** in the patient's home	- summary to the GP and home-care nurse at discharge
c) **call centre** for support and communication with the GP and home –care nurse/patient	- patient contacts GP, who refers patient to specialist
d) **individual self-management plans** for pharmacological and non-pharmacological intervention to manage stable COPD and exacerbations<br>- tools to monitor disease<br>- medication easily available in the patient's home to manage exacerbations	- occasionally an individual self-management plan is tailored

Figure 2 The difference between usual care in Trondheim, Norway and care using the COPD -Home model.

© 2013 Nordic College of Caring Science

Conclusions

COPD–Home combines elements of several models of integrated care reflecting the main principles: encouragement of patient self-management, a holistic approach to the patient, an effective response to the needs of the patient and shared accessible information (32). The COPD–Home model is an attempt to increase patients' and home-care nurses' competence and behaviours in accordance with guidelines for pharmacological and nonpharmacological interventions on a lifelong basis. The next step will be to evaluate whether such an integrated care model actually increases competence, actions, adherence to treatment, and improves the QOL and coping of patients with COPD.

Designing intervention programmes and evaluating their effects can contribute to the management of COPD and strengthen research-based care and treatment. Identifying the models that produce the best results in terms of reduced exacerbations, and improved QOL and coping can help both healthcare workers and planners to improve the management of COPD.

Acknowledgements

We thank all the project members from the municipality of Trondheim; Dr. Elena Titova at the Department of Thoracic Medicine, University Hospital of Trondheim; Hugh M. Allen language consultant; Carme Hernandez for her inspiration; InnoMed for advice on the layout of 'My COPD book'; Oldenburg Consulting for developing the e-learning programme; and the Central Norway Regional Health Authority for financial support.

Author contributions

Solfrid Jakobsen Lunde and Eva Marie Wangen participated in study conception and data collection and drafted the manuscript. Tone Rustøen and Signe Berit Bentsen drafted the manuscript and participated in critical revisions for important intellectual content and supervision. Rolf Axel Walstad initiated and participated in study conception. Anne Hildur Henriksen is responsible for and participated in study concept, drafted the manuscript and involved in supervision. Synnøve Sunde participated in study concept, data collection, drafted the manuscript and wrote the article.

Ethical approval

The study was approved by the Regional Ethical Committee.

Funding

The development of the COPD–model and later research is funded by the Central Norway Regional Health Authority.

References

1 GOLD. *Global Strategy for the Diagnosis, Management and Prevention of COPD, Global Initiative for Chronic Obstructive Lung Disease (GOLD)*. 2013, http://www.goldcopd.org/ (last accessed 15 March 2013).

2 Anzueto A. Impact of exacerbations on COPD. *Eur Respir Rev* 2010; 19: 113–8.

3 Connors AF Jr, Dawson NV, Thomas C, Harrell FE Jr, Desbiens N, Fulkerson WJ, Kussin P, Bellamy P, Goldman L, Knaus WA. Outcomes following acute exacerbation of severe chronic obstructive lung disease. The SUPPORT investigators (Study to Understand Prognoses and Preferences for Outcomes and Risks of Treatments). *Am J Respir Crit Care Med* 1996; 154: 959–67.

4 Liaaen ED, Henriksen AH, Stenfors N. A Scandinavian audit of hospitalization s for chronic obstructive pulmonary disease. *Respir Med* 2010; 104: 1304–9.

5 Carlson JE, Zocchi KA, Bettencourt DM, Gambrel ML, Freeman JL, Zhang D, Goodwin JS. Measuring frailty in the hospitalized elderly: concept of functional homeostasis. *Am J Phys Med Rehabil* 1998; 77: 252–7.

6 Creditor MC. Hazards of hospitalization of the elderly. *Ann Intern Med* 1993; 118: 219–23.

7 Bentsen SB, Rokne B, Wahl KW. Comparison of health-related quality of life between patients with chronic obstructive pulmonary disease and the general population. *Scand J Caring Sci* 2012; doi: 10.1111/scs.12002.

8 Gudmundsson G, Gislason T, Lindberg E, Hallin R, Ulrik CS, Brondum E, Nieminen MM, Aine T, Bakke P, Janson C. Mortality in COPD patients discharged from hospital: the role of treatment and co-morbidity. *Respir Res* 2006; 7: 109.

9 Griffiths P, Murrells T, Dawoud D, Jones S. Hospital admissions for asthma, diabetes and COPD: is there an association with practice nurse staffing? A cross sectional study using routinely collected data. *BMC Health Serv Res* 2010; 10: 276.

10 Griffiths P, Maben J, Murrells T. Organizational quality, nurse staffing and the quality of chronic disease management in primary care: observational study using routinely collected data. *Int J Nurs Stud* 2011; 48: 1199–210.

11 Ketelaars CA, Huyer Abu-Saad H, Halfens RJ, Schlosser MA, Mostert R, Wouters EF. Effects of specialized community nursing care in patients with chronic obstructive pulmonary disease. *Heart Lung* 1998; 27: 109–20.

12 Grone O, Garcia-Barbero M. Integrated care: a position paper of the WHO European Office for Integrated Health Care Services. *Int J Integr Care* 2001; 1: 21.

13 Koff PB, Jones RH, Cashman JM, Voelkel NF, Vandivier RW. Proactive integrated care improves quality of

© 2013 Nordic College of Caring Science

life in patients with COPD. *Eur Respir J* 2009; 33: 1031–8.

14 Bourbeau J, Nault D, Dang-Tan T. Reduction of hospital utilization in patients with chronic obstructive pulmonary disease: a disease-specific self-management intervention. *Arch Intern Med* 2003; 163: 585–91.

15 Casas A, Troosters T, Garcia-Aymerich J, Roca J, Hernandez C, Alonso A, del Pozo F, de Toledo P, Anto JM, Rodriguez-Roisin R, Decramer M, members of the CHRONIC Project. Integrated care prevents hospitalization s for exacerbations in COPD patients. *Eur Respir J* 2006; 28: 123–30.

16 Garcia-Aymerich J, Hernandez C, Alonso A, Casas A, Rodriguez-Roisin R, Anto JM, Roca J. Effects of an integrated care intervention on risk factors of COPD readmission. *Respir Med* 2007; 101: 1462–9.

17 Skwarska E, Cohen G, Skwarski KM, Lamb C, Bushell D, Parker S, MacNee W. Randomized controlled trial of supported discharge in patients with exacerbations of chronic obstructive pulmonary disease. *Thorax* 2000; 55: 907–12.

18 Shepperd S, Doll H, Broad J, Gladman J, Iliffe S, Langhorne P, Richards S, Martin F, Harris R. Hospital at home early discharge. *Cochrane Database Syst Rev* 2011; 21: CD000356.

19 Shepperd S, Iliffe S. Hospital at home versus in-patient hospital care. *Cochrane Database Syst Rev* 2005; 20: CD000356.

20 Pesut D, Ciobanu L, Nagorni-Obradovic L. Pulmonary rehabilitation in chronic respiratory diseases—from goals to outcomes. *Pneumologia* 2008; 57: 65–69.

21 Ergun P, Kaymaz D, Gunay E, Erdogan Y, Turay UY, Demir N, Canak E, Sengül F, Egesel N, Köse SK. Comprehensive out-patient pulmonary rehabilitation: treatment outcomes in early and late stages of chronic obstructive pulmonary disease. *Ann Thorac Med* 2011; 6: 70–76.

22 Nissen I, Jensen MS. Nurse-supported discharge of patients with exacerbation of chronic obstructive pulmonary disease. *Ugeskr Laeger* 2007; 169: 2220–3.

23 Hospital at Home. *Hospital Care in the Comfort Home*. 2012, http://www.hospitalathome.org (last accessed 22 January 2013).

24 Wong CX, Carson KV, Smith BJ. Home care by outreach nursing for chronic obstructive pulmonary disease. *Cochrane Database Syst Rev* 2011; 3: CD000994.

25 Bourbeau J, Nault D, Dang-Tan T. Self-management and behavior modification in COPD. *Patient Educ Couns* 2004; 52: 271–7.

26 Nici L, Donner C, Wouters E, Zuwallack R, Ambrosino N, Bourbeau J, Carone M, Celli B, Engelen M, Fahy B, Garvey C, Goldstein R, Gosselink R, Lareau S, MacIntyre N, Maltais F, Morgan M, O'Donnell D, Prefault C, Reardon J, Rochester C, Schols A, Singh S, Troosters T; ATS/ERS Pulmonary Rehabilitation Writing Committee. American Thoracic Society/European Respiratory Society statement on pulmonary rehabilitation. *Am J Respir Crit Care Med* 2006; 173: 1390–413.

27 Wilde MH, Garvin S. A concept analysis of self-monitoring. *J Adv Nurs* 2007; 57: 339–50.

28 NICE guidelines. *Chronic obstructive pulmonary disease- Management of chronic obstructive pulmonary disease in adults in primary and secondary care. Clinical guidelines*, CG 12. London: National Institute for Clinical Excellence, 2010: 1-53, http://www.nice.org.uk/CG012 (last accessed 22 January 2013).

29 Soumerai SB, Avorn J. Principles of educational outreach ('academic detailing') to improve clinical decision making. *JAMA* 1990; 263: 549–56.

30 Zakrisson AB, Hägglund D. The asthma/COPD nurses' experience of educating patients with chronic obstructive pulmonary disease in primary health care. *Scand J Caring Sci* 2010; 24: 147–55.

31 Hayes E, Kalmakis KA. From the sidelines: coaching as a nurse practitioner strategy for improving health outcomes. *J Am Acad Nurse Pract* 2007; 19: 555–62.

32 Escarrabill J. Discharge planning and home care for end-stage COPD patients. *Eur Respir J* 2009; 34: 507–12.

33 Morgan MD. Integrated care for COPD. What exactly do we mean? *Chron Respir Dis* 2008; 5: 131–2.

34 Simpson AC, Young J, Donahue M, Rocker G. A day at a time: care giving on the edge in advanced COPD. *Int J Chron Obstruct Pulmon Dis* 2010; 5: 141–51.

© 2013 Nordic College of Caring Science
