# Supplementary material for: Does an Integrated Care Intervention for COPD Patients Have Long-Term Effects on Quality of Life and Patient Activation? A Prospective, Open, Controlled Single-Center Intervention Study
Source: PLoS One. 2017 Jan 6;12(1):e0167887. doi: 10.1371/journal.pone.0167887 (PMC5218408; doi:10.1371/journal.pone.0167887)
Supplement: S3 Text — (DOC) [file pone.0167887.s003.doc]

**
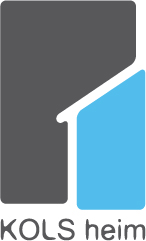
**

**PROSJEKTHÅNDBOK**

KOLS-HEIM prosjektet

Et samarbeidsprosjekt mellom

Trondheim Kommune, Avdeling Helse og Velferd og

St.Olavs Hospital HF, Lungeavdelingen

**Styringsgruppens sammensetning:**

- Anne Hildur Henriksen, overlege og klinikksjef ved Lungeavdelingen, St.Olavs Hospital, **prosjektansvarlig**
- Helge Garåsen, kommuneoverlege, Trondheim kommune
- Rolf Windspoll, samhandlingssjef St.Olavs Hospital
- Unni Dahl, Helse Midt – Norge
- Merete Rørvik, sekretariatsleder, InnoMed

**Arbeidsgruppens sammensetning:**

- Elin Eriksen, sykepleier Trondheim kommune
- Tove Røsstad, overlege Trondheim kommune
- Torgeir Fjermestad, overlege Trondheim kommune
- Jarl Reitan, Sintef InnoMed
- Kristine Holbø, Sintef InnoMed
- Solfrid Jakobsen Lunde, fagsykepleier Lungeavdelingen, St.Olavs Hospital (prosjektmedarbeider)
- Rolf Walstad, overlege Lungeavdelingen, St.Olavs Hospital (prosjektleder)
- Synnøve Sunde, fagansvarlig sykepleier Lungeavdelingen, St.Olavs Hospital (daglig prosjektleder/ prosjektkoordinator)

Endringer i prosjekthåndboka:

| Dato:13.06.08 | Dato: | Dato: | Dato: | Dato: |
| --- | --- | --- | --- | --- |
| Sign.:S.Sunde | Sign.: | Sign.: | Sign.: | Sign.: |

Trondheim, 2008-06-13 v.1.1

| **INNHOLD** |
| --- |

1. **PROSJEKTBESKRIVELSE**
   1. Innledning………………………………………………………………………….4
   2. Bakgrunn…………………………………………………………………………..4
   3. Mål………………………………………………………………………………….5
   4. Hypotese…………………………………………………………………………..5
   5. Begrepsavklaring………………………………………………………………....5
   6. Metode……………………………………………………………………………..5
   7. Inklusjon……………………………………………………………………………7
      1. Inklusjonskriterier……………………………………………………………..7
   8. Hjemmesykepleien………………………………………………………………..7
   9. Lungeavdelingen………………………………………………………………….7
   10. Fastlegen…………………………………………………………………………..8
   11. InnoMed………………………………………………………………..................8
   12. Teknisk utstyr……………………………………………………………………...8
   13. Evaluering…………………………………………………………………............8
       1. Endepunkter……………………………………………………………8
       2. Utvelgelse………………………………………………………………9
   14. Etiske aspekter, konfidensialitet og medisinsk ansvar………………………..9
   15. Prosjektorganisering…………………………………………………………… 10
       1. Styringsgruppe……………………………………………………… 10
       2. Arbeidsgruppe………………………………………………………...10
   16. Formidling………………………………………………………………………....10
   17. Nytteverdi………………………………………………………………………….11
       1. Kompetansespredning……………………………………………….11
       2. Reduserte kostnader ved behandling av kronisk syke…………...11
   18. Prosjektmiljøene………………………………………………………………….11
   19. Finansiering……………………………………………………………………….12
   20. Fremdriftsplan…………………………………………………………………….13

referanser

1. **PROTOKOLL**
   1. Flytskjema ………………………………………………………………………..14
   2. Studieplan…………………………………………………………………………16
   3. Pasientinformasjon og samtykkeerklæring……………………………………17
   4. Registreringsskjema…………………………………………………………… .17
      1. Inklusjon
      2. Pasientdata /registreringer
      3. Medikamenter ved utskrivelse
      4. Sjekkliste før utskrivelse
      5. plan for personlig oppfølging
   5. Spørreskjema……………………………………………………………………..18
      1. St.George´s Respiratory Questionnaire (SGRQ)
      2. Hospital Anxiety and Depression Scale (HADS)
      3. Patient Aktivation Measure (PAM)
   6. Observasjoner og registreringer i ”Min KOLS bok”…………………………18
   7. Medikamentadministrering…………………………………………………….19
   8. Kommunikasjon…………………………………………………………………20
   9. KOLS-sentral …………………………………………………………………...20
   10. Hjemmebesøk…………………………………………………………………...21
       1. Innhold………………………………………………………………..21
       2. Transport……………………………………………………………..21
   11. Kort for registrering av bruk av helsetjenester……………………………….21
   12. Dokumentasjon………………………………………………………………….21

**VEDLEGG**

Skjemabok

Min KOLS bok

Kort for registrering av bruk av helsetjenester

| 1. **PROSJEKTBESKRIVELSE** |
| --- |

- 1. **Innledning**

# Prosjektets mål er å utarbeide et system for hjemmebasert behandling, overvåkning, omsorg og rehabilitering av pasienter med alvorlig kronisk obstruktiv lungesykdom. Prosjektet vil legge vekt på bedret, mer aktiv og effektiv samhandling mellom profesjoner og nivåer i helsetjenesten, dvs. mellom kommune- og spesialisthelsetjenesten. Samtidig vil en legge forholdene til rette for bedre pasientmedvirkning. Endelig vil en kartlegge behovet for elektroniske verktøy for å effektivisere samarbeidet mellom de involverte parter. På denne måten vil en kunne gi et bedre helhetlig helsetilbud til pasientene, forbedre og kvalitetssikre det tilbudet de har i dag til lavere kostnader for samfunnet. Prosjektet er godkjent av Regional komité for medisinsk forskningsetikk og av Personvernsombudet for forskning.

”Samhandlingskjeden for kronisk syke” er et tilstøtende prosjekt, men i dette prosjektet vil KOLS-pasienter bosatt i Trondheim kommune utelates.

- 1. **Bakgrunn**

Pasienter med alvorlig KOLS og begynnende lungesvikt krever avansert behandling og oppfølging. Disse sykdommene rammer en stadig større andel av befolkningen og har direkte relasjon til røyking. Av landets 1.2 mil. røykere, har ca. 250.000 KOLS (1). Antall sykehusinnleggelser på grunn av KOLS var i 1995 over 15000. De fleste av disse (53 %) befant seg i aldersgruppen over 65 år, en aldersgruppe som vokser i antall.

Pasientene har som sine viktigste plager: tung pust ved anstrengelser/hvile og hoste og seigt slim som er vanskelig å få opp. Plagene er til stede hver dag, og er med på å redusere deres livskvalitet. I tillegg har de akutte anfall med forverringer, ofte mer enn 2 ganger/år. De med alvorlig og langt fremskreden sykdom kan ha 4 – 6 forverringer pr. år. Under anfallene blir pasientene alvorlig syke og må legges inn som øyeblikkelig hjelp i sykehus for avansert medisinsk behandling. Årsaken til de akutte forverringene er ofte virus – eller bakterieinfeksjoner i luftveiene, men de kan også utløses av reaksjoner på allergener, uspesifikke irritanter, klimaforandringer, luftforurensing, o.l. De hyppige forverringene med stadige og langvarige sykehusinnleggelser preger deres livssituasjon. I 1995 var den gjennomsnittlige liggetid for disse pasientene 7,9 dager, mens liggetiden ellers for en lungepasient var på 5,3 dager. Det må videre nevnes at disse pasientene i tillegg bruker 8 – 12 uker på å komme seg. Pasientgruppen tilhører Lungeavdelingens ”svingdørspasienter”, og er storforbrukere av helsetjenester. Ved Lungeavdelingen legges det inn 700 pasienter med KOLS-forverring, og halvparten er ”gjengangere”. Endelig er det i flere studier vist at KOLS pasienter med hyppige forverringer (> 2/år) har økt mortalitet og nedsatt livskvalitet. Overlevelse etter 5 år er på 40-50 %.

I tillegg er mange av disse pasientene preget av annen sykelighet, har kompliserende tilleggssykdommer, bruker mange medikamenter med til dels alvorlige bivirkninger og medikamentene er vanskelig å ta (inhalatorer). Alt dette medfører redusert psykisk, fysisk og sosial livskvalitet, og det kan påvirke deres familiære og økonomiske situasjon.

Det er derfor svært viktig at basisbehandlingen av denne pasientgruppen er optimal, og at en kan intervenere tidlig dersom de får symptomer på forverring.

I tillegg er lungerehabilitering (opplæring, mestring, fysioterapi, trening, kosthold, tilrettelegging osv.) og røykekuttprogram svært viktig.

- 1. **Mål**

Prosjektets hovedmål er å bedre den hjemmebaserte behandlingen av pasienter med alvorlig lungesykdom, slik at antall forverringer, sykehusinnleggelser og bruk av helsetjenester reduseres. Det er videre en målsetning at pasienten skal oppleve økt grad av mestring av egen situasjon og en bedret livskvalitet.

- 1. **Hypotese**

Bedret samhandling mellom nivåene i helsetjenesten vil ha betydning for den enkelte pasients fysiske og psykiske helse og medføre endringer i pasientens behov og forbruk av helsetjenester. Vår hypotese er at behovene reduseres og vil føre til helsegevinster for den enkelte og økonomiske gevinster for helsevesenet, Intervensjonen skal sammenliknes med en gruppe som får vanlig behandling.

- 1. **Begrepsavklaring**

Tjenester i hjemmet:

Hjemmetjenesten omfatter kommunale tjenester som, etter vedtak ved Helse – og velferdskontoret, tilbys personer som ønsker og kan bo i eget hjem. Tjenester i hjemmet inneholder følgende deltjenester 1) Hjemmesykepleie som er en del av kommunehelsetjenesten.. 2) Trygghetsalarm og praktisk bistand i hjemmet er sosiale tjenester ; som for eksempel, husvask, klevask og snømåking.

Hjemmesykepleien:

Hjemmesykepleien er en del av den kommunale helsetjenesten og bidrar med sykepleietjenester.

Ressurspersoner:

Ressurspersoner er de sykepleiere/ hjelpepleiere/ omsorgsarbeidere i Midtbyen og Østbyen som sonelederne har utpekt til å være ressurser i forhold til KOLS-heim prosjektet. Lungeavdelingen har gitt opplæring til hjemmesykepleiens ressurspersoner.

Prosjektsykepleiere:

Prosjektsykepleiere er erfarne sykepleiere ved Lungeavdelingen som har fått opplæring i KOLS-heim prosjektet.

- 1. **Metode**

Kompetansehevning: Bygge opp bedre kompetanse om behandling og omsorg av pasientgruppen i kommunehelsetjenesten. Kurs om KOLS sykdomslære, behandling og pleie. Medikamentlære, observasjon og registrering av symptomer i ”Min KOLS-bok”

Tettere oppfølging rundt og etter utskrivning:

- Forbedre utskrivningsrutinene ved Lungeavdelingen ved innføring av ”Min KOLS-bok” som skal følge den enkelte pasient. Den inneholder sentral informasjon om pasientens sykdom, lungefunksjonen, andre sykdommer, medikamenter, behandlingsplan med stående forordninger, observasjons- og registreringsskjema. Den skal til enhver tid være oppdatert og følge pasienten ved all kontakt med helsetjenesten.
- ADL (vha IPLOS) må registreres ved oppstart og evaluering
- Hjemmebesøk av prosjektsykepleier, ressursperson og fastlege samlet. Vi har valgt at det skal gjennomføres 2 slike besøk, fordi det er det vi anser gjennomførbart i forhold til tilgjengelige ressurser. Det første besøket innen tre dager etter utskrivelse og det andre innen 14 dager etter utskrivelse, eller først det lar seg organisere. Det er ønskelig at fastlegen deltar på det andre hjemmebesøket, *alternativt,* at pasienten kommer til kontroll hos fastlegen ca. 14 dager etter utskrivelsen. Det skal dokumenteres hvem som møter på hjemmebesøkene.
- Etablering av KOLS -sentral ved Lungepoliklinikken. Her mottas og registreres observasjonene som gjøres av hjemmesykepleien hos den enkelte pasient og overføres prosjektets database. De vurderes av prosjektsykepleier og ansvarlig lungelege. Beslutninger om ny behandlingsstrategi (medikamentjusteringer, henvisning til poliklinikk, undersøkelser, innleggelser i sykehus, etc.) kommuniseres så tilbake til hjemmesykepleien. Ved medikamentendringer melder hjemmesykepleien dette til fastlegen.
- All journalføring i GERICA skal fortsette som normalt og data hentes ut av pasientjournalen ved evaluering

Livsstilsintervensjon: Lungerehabiliteringen ved Lunge poliklinikk systematiseres og planlegges som en forlengelse av behandlingen ved Lungeavdelingen. Tilbudet er frivillig men det er en forventning om at pasienten takker ja til å delta på aktuelle tilbud.

1. Røykestopp kurs: Pasientene vil få tilbud om - og motiveres til å følge de planlagte røykestopp kursene som gjennomføres ved Lungepoliklinikk. Ansvarlig for gjennomføringen er sykepleier/lege ved poliklinikken.
2. Lærings- og mestringskurs: inneholder teoretisk og praktisk undervisning om sykdommene, medikamentene, administrasjonen av dem, treningslære, kosthold, trygderettigheter, tilrettelegging i heimen, mestring, å leve med KOLS osv. Aktivitetene er tverrfaglig (lege, sykepleier, fysioterapeut, ergoterapeut, likemann, ernæringsfysiolog, sosionom).
3. Fysioterapi/trening: Pasientene vil få tilbud om å delta individuelt og i de grupper som behandles poliklinisk av fysioterapeutene ved Lungeavdelingen, ved private institutter eller I kommunal regi.

Pasient, evt. pårørende, ressursperson og prosjektsykepleier oppretter i fellesskap en personlig plan for aktuell livsstilsintervensjon med utgangspunkt i den enkeltes nærmiljø. Hjemmesykepleien vil, dersom det er behov for det, benytte kommunehelsetjenestens (eks. ergoterapi) instanser for å gjøre pasientens hverdag lettere.

Alle disse elementene sees på som svært viktige for gjennomføringen av prosjektet.

Vi har av ressurshensyn valgt at den tradisjonelle ”Individuell Plan” ikke inngår som en del av prosjektet. Individuell plan vil allikevel være aktuelt for mange av deltagerne.

- 1. **Inklusjon**

Det er mulig å randomisere minimum 200 pasienter fordelt på 2 grupper.

Den ene gruppen vil bli randomisert til et intervensjonsopplegg som beskrevet ovenfor eller til en vanlig oppfølging og behandling i primærhelsetjenesten uten slik intervensjon. Randomiseringen vil skje etter den bydelen pasienten er bosatt. To av bydelene i Trondheim skal delta i prosjektet, mens pasienter fra to andre bydeler fungerer som kontrollgruppe. Det er ingen aldersbegrensning, og pasienter av begge kjønn kan delta.

En tar sikte på å inkludere 100 pasienter i hver gruppe.

Inklusjonstid: ca.1,5 år. Oppfølging: 2 år.

- - 1. **Inklusjonskriterier**
- bosatt i hjemmet i Trondheim kommune
- hjemmesykepleie minst x 1 pr.uke
- ikke lide av annen alvorlig sykdom med forventet levetid < 6 mndr.
- I stand til å gi informert samtykke
- KOLS grad 3 eller 4 (Gold kriterier)

Ved gateadresse i bydelene Heimdal eller Lerkendal: kontrollgruppe

Ved gateadresse i bydelene Østbyen eller Midtbyen: intervensjonsgruppe

- 1. **Hjemmesykepleien**

Hjemmesykepleien vil være sentral i prosjektet. De har til vanlig liten tradisjon og mulighet for løpende kontakt med sykehuset etter at pasienten er utskrevet. Det er ønskelig med et tettere samarbeid mellom omsorgsnivåene om denne pasientgruppen.

Ved kursing, hospitering og veiledning vil ressurspersonen i hjemmesykepleien få øket sin kompetanse om denne pasientgruppen. De vil få innføring i å observere, registrere, medisinere, behandle og gi omsorg til dem. De vil også få opplæring og trening i å bruke ”KOLS-boken” som vil bli et viktig hjelpemiddel i prosjektet. Videre vil det i samarbeidet med prosjektsykepleier ved KOLS-sentralen i Lunge poliklinikk bli gitt løpende veiledning underveis.

- 1. **Lungeavdelingen**

Lungeavdelingen ønsker å bedre behandlingen for pasienter med alvorlig lungesykdom slik at de unngår påkjenningene med stadige innleggelser (”svingdørspasienter”). Samtidig vil dette også redusere antallet korridorpasienter og presset på avdelingen. Videre ønsker en å bedre samhandlingen med kommunehelsetjenesten og bidra til økt kompetanse i egen avdeling og i kommunehelsetjenesten.

Det er etablert en koordinerende enhet ved Lungepoliklinikken, KOLS – sentral, som vil ha det overordnete prosjektansvar. Fagansvarlig sykepleier ved Lungeavdelingen er daglig prosjektleder/ prosjektkoordinator i 50 % st. og undervisningssykepleier er prosjektmedarbeider i 50 % stilling. Ved poliklinikken mottas og registreres observasjoner og data fra pasienten foretatt av hjemmesykepleien. Disse sammenholdes med pasientens behandlingsplan og journal, og vurderes av sykepleier og ansvarlig lege. Justering av behandlingen og andre tiltak kommuniseres tilbake til hjemmetjenesten som iverksetter tiltakene.

- 1. **Fastlegen**

På det andre hjemmebesøket, innen 14 dager etter utskrivelse, er det ønskelig at fastlegen deltar sammen med hjemmesykepleie og prosjektsykepleier. Alternativt bestiller pasienten selv kontroll hos sin fastlege ca. 14 dager etter utskrivelse og medbringer ”Min KOLS bok”. Fastlegen får brev vedlagt epikrisen om at pasienten deltar i prosjektet. Han må til enhver tid være oppdatert om pasientens tilstand, de diagnostiske og terapeutiske tiltak som gjennomføres i f. m. prosjektet. Dette ivaretas ved at hjemmesykepleien følger sine rutiner og varsler fastlegen om endringer i medikamentell behandling. Denne kommunikasjonen skal registreres i GERICA

- 1. **InnoMed**

Parallelt med det kliniske prosjektet skal InnoMed kartlegge behov og skissere hensiktsmessige løsninger for elektronisk kommunikasjon mellom de involverte miljøer.

Løsningene som vurderes skal være i tråd med vedtatte nasjonale IT –strategier. Målet er å komme fram til en elektronisk registrering av observasjoner i pasientens hjem og elektronisk overføring av data. Det foreligger en egen protokoll for denne studien (se vedlegg; Prosjektsøknad, side 8, pkt.2-Forprosjekt teknologi).

- 1. **Teknisk utstyr**

Det tekniske utstyret som er anskaffet i f. m. prosjektet er som følger:

1. Mobiltelefon/SMS for kommunikasjon mellom de samarbeidende enhetene.
2. PIKO 1 (enkelt spirometer)
3. PC for ajourføring og lagring av registrerte pasientdata, behandlingstiltak, observasjoner og plan for personlig livsstilsintervensjon
   1. **Evaluering**

Deler av prosjektet skal evalueres som ledd i en mastergrad i helsevitenskap. Det arbeides også med å få til et dr.grad stipend for studien. Resultatene skal publiseres og presenteres anerkjente nasjonale og internasjonale tidsskrifter.

Evalueringen vil bestå av en sammenligning mellom de to randomiserte gruppene m/u intervensjon.

- - 1. **Endepunkter**

Primære endepunkter vi være:

1. Antall sykehusinnleggelser med hoveddiagnose KOLS
2. Antall sykehusinnleggelser med annen hoveddiagnose
3. Antall liggedøgn

4) Livskvalitet målt ved livskvalitetsskjemaene.

5) Forbruk av hjemmesykepleie og andre kommunale helsetjenester (antall besøk

og tidsbruk hentes fra GERICA)

1. Forbruk av sykehjem (langtids og korttidsopphold) og dager før langtidsopphold

7) Død

Sekundære endepunkter vil være:

1. Fall i FEV1 og FVC
2. 6 min. gangtest og øvrige respirasjonsfysiologiske parametre
3. Antall konsultasjoner og sykebesøk av fastlegen
4. Antall corticosteroid – og antibiotikakurer
5. Behov for ventilasjonsstøtte og overlevelse
6. ADL (IPLOS)
7. Antall besøk av legevakt (hentes fra…..)

Tertiære endepunkter vil være en komparativ kostnads – nytte analyse av effekten av intervensjonen:

1. prosjektkostnader (opplæring og drift)
2. kostnader pga. endret behov/ forbruk av helsetjenester

(spesialist - og primær/kommunehelsetjeneste)

1. tilleggsverdier (pasienttilfredshet, samhandlingsverdi)
2. Medikamentbruk

Endepunktene måles etter 1, 2, og 3 år og sammenlignes med data fra kontrollgruppen. Det er ønskelig med en evaluering også ved 6 måneder. Det vi bli avgjort senere om det er nok tilgjengelige ressurser til å gjennomføre dette. Enkle komparative statistiske metoder skal benyttes. Studiens design er åpen, men kontrollert og randomisert.

Vi vurderte at vi ikke har kapasitet til å innhente pålitelige data for mengde ikke kommunale tjenester og uformell hjelp i hjemmet, noe som også kan variere veldig i løpet av en 3 års periode. Dette vi derfor ikke bli evaluert.

- - 1. **Utvelgelse**

Den kommunale helsetjenesten i Trondheim er inndelt i 4 bydeler. Det er foretatt en parvis matching av bydelene med den største (Lerkendal) og den minste (Heimdal), til sammen ca. 83 000 innbyggere, mot det andre paret (Østbyen og Midtbyen) som til sammen har en befolkning på ca. 75 000. Befolkningssammensetningen i bydelene er noenlunde likt fordelt i alder og sykdomspanorama. Antall innbyggere med alder hvor KOLS dominerer (55 – 79 år) er lik i de to gruppene, henholdsvis ca.15 800 (kontroll; Lerkendal / Heimdal) og 15 200 (intervensojn; Østbyen / Midtbyen). Det er ved loddtrekning bestemt at det skal interveneres i Østbyen / Midtbyen. Randomiseringen av pasientene kan derfor skje etter den bydelen de er bosatt (adresse).

I de to bydelene i Trondheim som skal delta i prosjektet vil hjemmetjenesten få kurs og opplæring i KOLS og den intervensjonen som skal gjennomføres. De andre to bydelene får intet slikt tilbud. Det er ingen aldersbegrensning og pasienter av begge kjønn kan delta.

Det innlegges ca. 700 pasienter med akutt forverring av KOLS på år, ca. 600 fra Trondheim, og halvparten er ”gjengangere”. En kan derfor regne med å kunne inkludere 100 pasienter i hver gruppe. Med 100 pasienter i hver gruppe (totalt 200 pasienter) vil det være 85% sannsynlighet for å påvise 20% forskjell mellom gruppene i effektmålene (alfa 0,05; beta 0,20).

- 1. **Etiske aspekter, konfidensialitet og medisinsk ansvar**

Prosjektet er godkjent av Komiteen for medisinsk forskningsetikk, Helseregion IV. Pasientene vil få både skriftlig og muntlig informasjon om prosjektet og må underskrive en samtykkeerklæring. De vil også bli informert om at de kan trekke seg fra prosjektet når som helst uten å gi noen begrunnelse. De som er i kontrollgruppen og de som trekker seg eller ikke ønsker å delta, vil bli behandlet og fulgt opp akkurat som i dag både i sykehus og kommune. Intervensjonsgruppen får i prinsippet ikke ny eller annen behandling, men en tettere oppfølging etter utskrivelsen fra sykehuset.

Alle prosjektmedarbeidere må skrive under på erklæring om taushetsplikt dersom de ikke har gjort det før.

Det overordnete medisinske ansvar for prosjektet vil ligger hos klinikksjefen ved Lungeavdelingen, St. Olavs Hospital

- 1. **Prosjektorganisasjonen**

Prosjektet er organisert med en styringsgruppe, en arbeidsgruppe, en daglig prosjektleder/ prosjektkoordinator i 50 % stilling og en prosjektmedarbeider i 50 % stilling.

- - 1. **Styringsgruppen**

Styringsgruppen har det formelle ansvaret for prosjektet og tar de endelige avgjørelsene vedrørende arbeidsprosesser og løsninger av strategisk betydning.

### Ansvar og oppgaver

- Ansvar for oppstart av prosjektet
- Kan stoppe prosjektet ved store avvik som død eller vesentlig økt bruk av sykehustjenester på intervensjonsgruppen
- Skal kontrollere at prosjektplan og budsjett følges
- Godkjenner prosjektets resultat
- Tar beslutninger om avvikshåndtering
- Skal mobilisere organisasjonene og tilrettelegge for at samarbeidet mellom organisasjonene fungerer
- Skal godkjenne forslag til nye arbeidsmetoder
  - 1. **Arbeidsgruppen**

Arbeidsgruppen leder prosjektets driftsorganisasjon. Gruppen har ansvaret for utarbeidelse av prosedyrer, opplæring, kvalitetssikring, og rapporterer til styringsgruppen. De er budsjettansvarlige, og har ansvar for samarbeidsprosessene mellom partene/nivåene.

Arbeidsgruppa er ansvarlig overfor styringsgruppa i forhold til prosjektets gjennomføring og resultat.

Daglig prosjektleder/ prosjektkoordinator er medlem av arbeidsgruppen og styrer den daglige driften av prosjektet.

- 1. **Formidling**

Siden prosjektet er en kombinasjon av brukerrettet modellutprøving og produkt/tjenesteutvikling vil formidlingen bestå av følgende:

- opplæring av helsepersonell i bruk av utstyr/arbeidsmetoder
- presentasjoner i konferanser og brukermøter
- rapporter, vitenskapelige og populærvitenskapelige publikasjoner og presentasjoner, nasjonalt og internasjonalt
- dekning i nasjonale media

(se også evaluering, pkt.1.12)

- 1. **Nytteverdi**
     1. **Kompetansespredning**

Prosjektet vil heve kompetansen om pasienter med alvorlig lungesykdom i primærhelsetjenesten. Dette vil også bedre spesialisthelsetjenestens forståelse for de problemstillinger som er sentrale primærhelsetjenesten. Pasientene og deres pårørende vil også få økte kunnskaper, bedre mestring og økt trygghet gjennom prosjektet. Hjemmesykepleiens ressurspersoner vil få teoretisk og praktisk innføring i den nye behandlingsmetoden for kronisk lungesyke (jmfr. Opplæringsplan). Prosjektet vil også, dersom det blir vellykket, kunne videreføres lokalt og til andre miljøer i Norge.

Gjennom prosjektet får pasientene et bedret hjemmebasert tilbud med muligheter til å intervenere tidligere. Større trygghet med færre innleggelser og bedret livskvalitet vil kunne oppnås. Tilbudet om rehabilitering vil gir pasientene et komplett og helhetlig behandlingstilbud.

- - 1. **Reduserte institusjonskostnader ved behandling av kronisk syke**

**pasienter**

Hypotesen er at de nye hjemmebaserte behandlingsmetodene vil føre til mindre press på Lungeavdelingens sengeposter og institusjonsplasser i Trondheim kommune. Det kan bli færre korridor- og svingdørspasienter. Det kan bli billigere å behandle pasienten i hjemmet, intervenert tidligere og forhindre sykehusinnleggelse og utsette behovet for langtids sykehjemsplass. Gjennom prosjektet vil pasienten og pårørende få økte kunnskaper om egen sykdom, og dette vil også kunne føre til økt trygghet og færre innleggelser.

Videre vil samarbeidet mellom kommune- og spesialisthelsetjenesten bli bedre og mer integrert.

- 1. **Prosjektmiljøene**

Prosjektgruppen består av 3 miljøer, som hver er bærere av kompetanse som er nødvendig for å gjennomføre prosjektet.

Lungeavdelingen, St.Olavs Hospital

Lungeavdelingen på St.Olavs Hospital omfatter poliklinikk, 36 døgnsenger, 4 dagplasser og 3 overvåkningssenger og tar seg blant annet av de kronisk syke lungepasientene. Behandlingstilbudet for disse pasientene består av medisinering, oksygenbehandling og

opplæring av forskjellig slag. Lungeavdelingen har erfaring både i poliklinisk og institusjonsbasert lungerehabilitering.

Lungeavdelingen ønsker seg en mer effektiv behandling av pasienter med alvorlig lungesykdom og mener veien å gå er gjennom en bedre kommunikasjon med 1. linjetjenesten, pasienten selv, pårørende og pasientenes interesseorganisasjoner.

## Trondheim kommune, Hjemmetjenesten

I Trondheim kommune er det 4 servicekontor for helse og omsorgstjenester lokalisert i forskjellige bydeler. Hjemmetjenestens kontorer skal sikre alle innbyggerne lik tilgang på helse og omsorgstjenester uavhengig av alder, tjenestebehov og bosted i Trondheim. Alle som mener de har behov for hjelp, kan ta kontakt med kontoret i det området de bor i.
Trondheim kommune ønsker gjennom prosjektet å opparbeide seg kunnskap om samhandling med spesialisthelsetjenesten, og å bedre sin kompetanse om behandlingen av alvorlig syke lungepasienter. Et overordnet mål er også å bedre omsorgen for disse pasientene.

InnoMed vil gjennomføre IKT- pilotprosjekt i f.m. den kliniske intervensjonen. (se egen protokoll)

- 1. **Finansiering**

Helse Midt-Norge støttet prosjektet med kr. 400 000 i 2007 og kr. 800 000 i 2008.

Kr.183 000 overføres fra 2007 til 2008.

Innovasjon Norge støtter IKTdelen med kr. 200 000. Det vises til egen protokoll.

Budsjettet inkluderer timekostnad, samt timekostnader og dirkete kostnader for St. Olavs Hospital og Kommunehelsetjenesten.

|  | **2007** | | **2008** |
| --- | --- | --- | --- |
|  | **Prosjekt 1** | **Prosjekt 2** | **Prosjekt 1** |
| **St. Olavs Hospital** |  |  |  |
| Egeninnsats | 250 000,- | 50 000,- | 250 000,- |
| Lønnskostnader prosjektsykepleiere | 185 000,- |  | 520 000,- |
| Frikjøp av lege |  |  | 250 000,- |
| Frikjøp av sykepleier |  |  | 80 000,- |
| Utstyr / drift | 32 000,- |  | 30 000,- |
| **Trondheim Kommune** |  |  |  |
| Frikjøp av sykepleier |  |  | 100 000,- |
| Egeninnsats | 100 000,- | 50 000,- | 250 000,- |
| **InnoMed** |  |  |  |
| Prosjektarbeid | 100 000,- | 200 000,- |  |
| **Egeninnsats totalt** | 217 000,- |  | 500 000,- |
| **Bevilget** | 217 000,- | 300 000,- | 983 000,- |
| **Eg. Innsats + bevilget T** | **667 000,-** | **300 000,-** | **1 483 000,-** |

- 1. **Fremdriftsplan**

2007 2008 2009

|  | 9 | 10 | 11 | 12 | 1 | 2 | 3 | 4 | 5 | 6 | 7 | 8 | 9 | 10 | 11 | 12 | 1 | 2 | 3 | 4 | 5 | 6 | 7 | 8 | 9 | 10 | 11 | 12 |
| --- | --- | --- | --- | --- | --- | --- | --- | --- | --- | --- | --- | --- | --- | --- | --- | --- | --- | --- | --- | --- | --- | --- | --- | --- | --- | --- | --- | --- |
| For-prosjekt |  |  |  |  |  |  |  |  |  |  |  |  |  |  |  |  |  |  |  |  |  |  |  |  |  |  |  |  |
| Pilot |  |  |  |  |  |  |  |  |  |  |  |  |  |  |  |  |  |  |  |  |  |  |  |  |  |  |  |  |
| Hoved-prosjekt |  |  |  |  |  |  |  |  |  |  |  |  |  |  |  |  |  |  |  |  |  |  |  |  |  |  | * |  |

***** hovedprosjektet vil pågå til det er inkludert min.100 pasienter i hver av gruppene., anslagsvis 1,5 år. Evaluering ½,1, 2 og 3 år etter inklusjon.

**Referanser**

1. Gulsvik A, *Obstruktiv lungesykdom i sykehus*, Tidsskrift for den Norske Lægeforeningen, 1997
2. World Health Organization, Innovative Care for Chronic Conditions, WHO 2002
3. Gulsvik A, Lungesykdommer på fremmarsj - en epidemi som krever større årvåkenhet, Tidsskrift for den Norske Lægeforeningen, 1998
4. Myhre KI. *Telemedicine and health technology assessment*. Tidsskrift for den Norske Lægeforening, 19/2000.
5. Tjora, A.H. (2000) The Technological Mediation of the Nursing-Medical Boundary, *Sociology of Health & Illness,* vol 22, no 6, 2000, pp. 721-741.
6. Tjora, A.H (2001) Helsefabrikken - en fabrikkmetafor på sykehuset, i *Maskinkultur - Utsnitt fra fabrikkens tidsalder*, nr 3 i skriftserie fra prosjektet "Fabrikken" NTNU, mars 2001.
7. National Heart, Lung and Blood Institute & World Health Organisation: Global Initiative for Chronic Obstructive Lung Disease, Internet: [www.goldcopd.com](http://www.goldcopd.com/), April 2003

| 1. **PROTOKOLL** |
| --- |

- 1. **Flytskjema : under sykehusoppholdet (Lungeavdelingen/ Observasjonsposten)**

| **Registreringsskjema - Inklusjon**  Fyller pas. inklusjonskriteriene?  Hvis ”ja”: intervensjonsgruppe / kontrollgruppe?  - forutsetter spirometriverdier | Prosjektsykepleier vurderer daglig nyinnlagte pasienter |
| --- | --- |

| **Informasjon av pasient** | Når samtykkeerklæring er underskrevet er pasienten |
| --- | --- |
| **Samtykkeerklæring** | inkludert |
| **Spørreskjema ; SGRQ, HADS og PAM** | Fylles ut av pasient under sykehusoppholdet eller hjemme |

| **Registreringer i ”Min KOLS- bok”**  - forutsetter blodgassverdier |  |
| --- | --- |
| **Opplæring PIKO 1 (minispirometer)** | Prosjektsykepleier |

| **Utskrivningssamtale inkluderte;**   - **sjekkliste før utskrivning** | Lege  Pasient / pårørende  Prosjektsykepleier |
| --- | --- |

**Flytskjema etter sykehusoppholdet**

| **ADL** vha IPLOS registreres / oppdateres ved utreisetidspunkt | Ressursperson (hjemmesykepleien) |
| --- | --- |

| **1. hjemmebesøk innen 3 dager etter utskrivelse,** eller først det lar seg organisere   - faste observasjoner - registrere observasjoner i ”Min KOLS-bok” - Lage personlig oppfølgingsplan for livsstilsintervensjon | Pasient, evt. pårørende ressursperson og prosjektsykepleier |
| --- | --- |

| **2. hjemmebesøk innen14 dager etter utskrivelse,** eller først det lar seg organisere vurdert etter omstendigheter og behov  • oppfølging av 1. hjemmebesøk | Pasient, evt. pårørende, fastlege* ressursperson og  prosjektsykepleier |
| --- | --- |

*når fastlege deltar på hjemmebesøket *kan* dette erstatte kontroll hos fastlege etter ca.

14 dager (vurderes av fastlegen)

| **Kontroll hos fastlege;** ca. 14 dager etter utskrivelse | Pasienten bestiller selv time  ”Min KOLS bok” medbringes |
| --- | --- |

| **Kontinuerlig**   - Vanlig oppfølging etter vedtak og journalføring i GERICA - Faste observasjoner listet i ”Min KOLS bok” - Registrering på Observasjonsarket i ”Min KOLS-bok” | Hjemmesykepleien |
| --- | --- |

| **Utfylt observasjonsark (fullt ark)** | Oppbevares i ”Min KOLS-bok” i 1 måned, sendes deretter KOLS-sentralen, adresse side19 |
| --- | --- |

| **Etter ½ år - 1 år - 2 år – 3 år**   - Nye registreringer ved KOLS-sentralen - ADL oppdateres | Prosjektsykepleier  Hjemmesykepleien |
| --- | --- |

- 1. **Studieplan**

|  | **Innleggelse**  **Lungeavd.** | **1.hjemmebesøk** | **2.hjemmebesøk** | **Etter:**  **½ år** | **1år** | **2år** | **3år** |
| --- | --- | --- | --- | --- | --- | --- | --- |
| **Dato** |  |  |  |  |  |  |  |
| **Informere pasient I/K** | **X** |  |  |  |  |  |  |
| **Samtykkeerklæring I/K** | **X** |  |  |  |  |  |  |
| **Inkludering /ekskludering** | **X** |  |  |  |  |  |  |
| **Reg.skjema-Pasientdata I/K** | **X** |  |  | **X** | **X** | **X** | **X** |
| **SGRQ I/K** | **X** |  |  | **X** | **X** | **X** | **X** |
| **HAD I/K** | **X** |  |  | **X** | **X** | **X** | **X** |
| **PAM I/K** | **X** |  |  | **X** | **X** | **X** | **X** |
| **Reg.skjema-Medikament I/K** | **X** |  |  | **X** | **X** | **X** | **X** |
| **ADL** vha IPLOS **I/K** | **X** |  |  | **X** | **X** | **X** | **X** |
| **Sjekkliste før utskrivelse I** |  |  |  |  |  |  |  |
| **Andre registreringer I/K** |  |  |  |  |  |  |  |
| FVC | **X** |  |  | **X** | **X** | **X** | **X** |
| FEV1 | **X** |  |  | **X** | **X** | **X** | **X** |
| FEV1/FVC | **X** |  |  | **X** | **X** | **X** | **X** |
| KOLS grad (Gold) | **X** |  |  | **X** | **X** | **X** | **X** |
| Ph | **X** |  |  | **X** | **X** | **X** | **X** |
| pCO2 | **X** |  |  | **X** | **X** | **X** | **X** |
| pO2 | **X** |  |  | **X** | **X** | **X** | **X** |
| Sat O2 | **X** |  |  | **X** | **X** | **X** | **X** |
| Høyde | **X** |  |  | **X** | **X** | **X** | **X** |
| Vekt | **X** |  |  | **X** | **X** | **X** | **X** |
| BMI | **X** |  |  | **X** | **X** | **X** | **X** |
| LTOT (min 14 t/døgn) | **X** |  |  | **X** | **X** | **X** | **X** |
| LTOT (kun v. fysisk aktivitet) | **X** |  |  | **X** | **X** | **X** | **X** |
| Røykevaner | **X** |  |  | **X** | **X** | **X** | **X** |
|  |  |  |  |  |  |  |  |
| **Min KOLS bok I** |  |  |  |  |  |  |  |
| Almentilstand fysisk | **X** | **X** | **X** |  |  |  |  |
| Almentilstand psyk. | **X** | **X** | **X** |  |  |  |  |
| Tungpust | **X** | **X** | **X** |  |  |  |  |
| Hoste | **X** | **X** | **X** |  |  |  |  |
| Oppspytt (mengde/farge) | **X** | **X** | **X** |  |  |  |  |
| Temperatur | **X** | **X** | **X** |  |  |  |  |
| Puls | **X** | **X** | **X** |  |  |  |  |
| Resp.frekvens | **X** | **X** | **X** |  |  |  |  |
| PEF | **X** | **X** | **X** |  |  |  |  |
| FEV1 | **X** | **X** | **X** |  |  |  |  |
| **Personlig plan I** |  | **X** | **X** | **X** | **X** | **X** | **X** |

**I= INTERVENSJONGRUPPEN**

**K=KONTROLLGRUPPEN**

- 1. **Pasientinformasjon og samtykkeerklæring**

Ved innleggelse på Lungeavdelingen eller Observasjonsposten skal pasienter som fyller inklusjonskriteriene (benytt eget skjema –”inklusjon”), informeres om prosjektet av prosjektsykepleier og gies god tid til å ta stilling til om han/ hun ønsker å delta i prosjektet. Det er utarbeidet 2 versjoner av pasientinformasjonen, én for de med adresse i bydelene Heimdal/ Lerkendal (kontrollgruppen) og én litt mer omfattende for de med bostedsadresse Østbyen / Midtbyen (intervensjonsgruppen).

Informert samtykkeerklæring skal utfylles i 2 eksemplarer. Pasienten beholder det ene(kopi) og det andre eksemplaret (originalen) oppbevares i pasientens skjemabok. Når samtykkeerklæringen er underskrevet er pasienten inkludert i studien.

- 1. **Registreringsskjema**

Det er utarbeidet mal for skjemabok, hvor alle skjema som skal benyttes i prosjektet er plassert. Det skal opprettes en bok pr. deltager. Denne skal oppbevares forskriftsmessig ved Lungeavdelingen.

- - 1. Inklusjon

Skjemaet benyttes til å sortere hvilke pasienter som fyller kriteriene til

deltagelse i prosjektet og om kandidatene skal til kontrollgruppen eller

intervensjonsgruppen. Grad av KOLS avgjøres ved spirometri. Hvis pasienten ikke

har spirometriresultater nyere enn 12 mndr, må ny spirometri tas maks 1 dag før

utskrivelse eller så nær utreisedato som mulig.

- - 1. Pasientdata /registreringer

Før utreise skal prosjektsykepleier fylle ut skjema for ”pasientdata/ registreringer”. Utfyllingen forutsetter at arteriell blodgass foreligger. Siste blodgass før utreise benyttes. Hvis blodgass ikke foreligger eller kun blodgass fra innkomst, må arteriell blodgass tas før utreise.

- - 1. Medikamenter ved utskrivelse

Etter utskrivningssamtalen skal prosjektsykepleier/utskrivende lege fylle ut (avkryssing) skjemaet ”medikamenter ved utreise”.

- - 1. Sjekkliste før utskrivelse

Utskrivende lege og prosjektsykepleier skal i utskrivningssamtalen gjennomgå skjemaet ”sjekkliste før utskrivelse”. (for utdypende informasjon; se i protokollen ang. de enkelte punkter på sjekklista)

- - 1. Plan for personlig oppfølging

”Plan for personlig oppfølging” skal lages i samarbeid mellom pasient/ pårørende, ressursperson og prosjektsykepleier ved første og/ eller andre hjemmebesøk. Prosjektsykepleier skal gjennomgå planen ved de polikliniske kontroller. Hjemmesykepleien skal kontinuerlig motivere pasienten for at planen skal følges opp og evt. bidra med enkel tilrettelegging/ søknader om vedtak/rettigheter.

- 1. **Spørreskjema**

I løpet av oppholdet på sykehuset skal pasienten motta 3 spørreskjema. Pasienten kan fylle ut skjemaene i løpet av oppholdet hvis tilstanden tilsier dette. Det er også greit at pasienten tar med skjemaene hjem og fyller ut hjemme i fred og ro. Prosjektsykepleier veileder i utfyllingen og tar inn skjemaene ved første hjemmebesøk eller senest ved andre hjemmebesøk. Ved kontroll etter ½, 1, 2 og etter 3 år skal de samme skjema benyttes.

- - 1. St.George´s Respiratory Questionnaire (SGRQ)

SGRQ er mye brukt I KOLS studier. Skjemaet er utviklet av Dr. P. W. Jones, MD, St.George´s Hospital Medical School, Dept. of Medicine, London og oversatt til norsk av en arbeidsgruppe nedsatt av Norsk Forening for Lungemedisin.

Skjemaet vil si noe om hvordan pasienten opplever sin livskvalitet relatert til lungesymptomer.

- - 1. HADS (Hospital Anxiety and Depression Scale)

HADS sier noe om pasientens opplevelse av sin livskvalitet relatert til emosjonell tilstand.

- - 1. Patient Activation Measure (PAM)

PAM er utviklet av Judith Hibbard, University of Oregon, USA og oversatt til norsk av Aslak Steinsbekk, Institutt for Samfunnsmedisin, NTNU. Skjemaet sier noe om i hvilken grad pasienten opplever å mestre sin situasjon.

- 1. **Observasjoner og registreringer i ”Min KOLS bok”**

Prosjektsykepleier/ utskrivende lege skal sørge for at s.1, s.2 og Observasjonsskjemaet blir fylt ut før første hjemmebesøk. Behandlingsplan med stående forordninger på for eksempel økt inntak av inhalasjonsmedisiner, antibiotika og / prednisolonkur, skal fylles ut av utskrivende lege og kommuniseres til pasienten i utskrivningssamtalen. Behandlingsplanen skal kvalitetssikres av ansvarlig lege. Prosjektsykepleier overleverer ”Min KOLS Bok” til pasienten ved første hjemmebesøk.

Ved første hjemmebesøk skal ressursperson og prosjektsykepleier i fellesskap foreta observasjoner og føre registreringer på Observasjonsskjemaet.

Videre skal hjemmesykepleien foreta observasjonene listet opp på Observasjonsskjemaet og daglig registrere på skjemaet inntil 2.hjemmebesøk fra prosjektsykepleier. Deretter skal observasjonsskjemaet fylles ut daglig ved det minste tegn på forverring og ellers minimum 1 gang pr.uke.

Når én eller flere registreringer kommer på gult eller rødt felt kan det være tegn på at brukeren har en begynnende forverring/ forverring av sin KOLS. Hjemmesykepleier må da samholde registreringene på Observasjonsskjemaet med Behandlingsplanen på s.4 – og følge denne.

Ved tvil om hvordan dette skal gjøres/ ønske om støtte på egne vurderinger skal hjemmesykepleier ringe KOLS-sentralen (prosjektsykepleier med lege ”back-up” på Lungeavdelingen) på tlf.nr. **48 23 00 66**, kl.08.30 – 15.30, alle hverdager. **Til øvrige tidspunkt skal hjemmesykepleier forholde seg til kommunens gjeldende retningslinjer.**

Ved alle forverrelser/ endret medisinering skal hjemmesykelpeien informere prosjektsykepleier ved ”KOLS-sentralen”.

Det er ett observasjonsskjema per måned. Ferdig utfylt skjema skal oppbevares bakerst i ”Min KOLS Bok ” i én måned og deretter sendes til;

Lungeavdelingen,

v/ sykepleier KOLS-HEIM prosjektet

St.Olavs Hospital, 7006 Trondheim

”Min KOLS Bok” skal følge pasienten ved kontakt med primærlege, poliklinikkbesøk og sykehusinnleggelser.

- 1. **Medikamentadministrering**

Multidosemedisinen pakkes ved at fastlegen sender sin liste med faste medikamenter til apoteket, som sender videre til pakkefirmaet. Multidoseliste datert og signert av legen gjelder som resept for ett år. Derfor skal **alle** pasientens medisiner være oppført på denne listen, også inhalasjoner og annet som ikke kan pakkes, samt medikamenter til evt. bruk.

Fordi fastlegens liste er grunnlaget for pakking må medikament-forandringer som gjøres av andre enn fastlegen straks formidles til fastlegen, som da fakser korrigert liste til apoteket.

I KOLS-heim prosjektet vil det for en del pasienter være aktuelt å bruke ”stående forordninger”, dvs at pasienten har tilgjengelig medisin, som skal brukes slik legen har bestemt når en sykdomstilstand utvikler seg. (om medisinen er tilgjengelig via resept eller om resepten er utløst og medisinen finnes i hjemmet kan vurderes i hvert tilfelle).

De symptomer og funn som utgjør kriteriene for å starte med medikamentet, skal foreligge skriftlig i ”Min KOLS-bok” og skal være presise og mest mulig objektive. Når kriteriene utvetydig er oppfylt kan sykepleier\vernepleier i Hjemmetjenesten iverksette medisineringen. Ved tvil skal lege (Lungeavd, fastlege, legevakt) først kontaktes.

Ved alle ferverrelser/ endret medisinering skal hjemmesykepleien informere prosjektsykepleier ved ”KOLS-sentralen”.

- 1. **Kommunikasjon**

Hjemmesykepleien

- gir etter gjeldende retningslinjer beskjed til fastlege ved medikamentendring
- kontakter prosjektsykepleier ved KOLS- sentralen for drøfting av endringer i behandling: telefon 48 23 00 66
- sender observasjonsskjemaet i ”Min KOLS bok” til prosjektsykepleier ved KOLS –sentralen i vanlig post
- informerer prosjektsykepleier ved ”KOLS – sentralen ved medikamentendring /forverrrelser

Lungeavdelingen

- sender etter gjeldende retningslinjer epikrise til fastlegen. I tillegg sendes brev som informerer om at pasienten er inkludert i prosjektet til fastlege og hjemmesykepleie.
  1. **KOLS-sentral**

**Telefonnummer: 48 23 00 66**

Telefonen er betjent av prosjektsykepleier alle hverdager fra kl. 08.30 – 15.30.

Lungelege er tilgjengelig i samme periode.

KOLS-sentralen ligger i gamle lungerøntgen, 1. etg., Lungebygget, St.Olavs Hospital.

Opprettelse av en KOLS-sentral er et av tiltakene i KOLS-HEIM prosjektet.

Direktetelefonen er et tilbud til hjemmetjenesten og kan benyttes ved problemstillinger

knyttet til behandling / oppfølging av pasienter som er inkludert i KOLS-HEIM prosjektet.

- 1. **Hjemmebesøk**

Prosjektsykepleier skal avtale det 1.hjemmebesøk med pasient, evt. pårørende og ressursperson, innen 3 virkedager etter utskrivelse fra sykehuset.

2.hjemmebesøk skal skje innen 14 dager etter utskrivelse, vurdert etter omstendigheter og behov..

Besøkene legges helst til slutten av dagen.

- - 1. **Innhold**

Ved hjemmebesøkene skal man i samarbeid foreta observasjoner listet opp i ”Min KOLS Bok” og registrere disse. Deretter enes om en personlig oppfølgingsplan. Innhold og bruk av Min KOLS bok skal presenteres og boka overleveres pasienten.

Spesielt fokus på behandlingsplan, observasjonsskjema.

Det må vurderes individuelt hvor mye pasienten orker å gjennomgå ved hvert av

besøkene

- - 1. **Transport**

Prosjektsykepleier skal benytte det fremkomstmiddel som er mest hensiktsmessig i forhold til tidsbruk og kostnader. Når pasienten bor utenfor gang-/sykkelavstand, benyttes egen bil, buss eller taxi. Kvitteringer beholdes og reiseregning belastes prosjektet.

- 1. **Kort for registrering av bruk av helsetjenesten**

Kortet er en del av ”Min KOLS bok ”. Kontrollgruppen mottar ikke ”Min KOLS bok”, men et eget kort for registreringer av bruk av helsetjenesten. Prosjektsykepleier har ansvar for innsamling av kortene og registrering i prosjektets database.

- 1. **Dokumentasjon**

Hjemmesykepleien skal dokumentere på vanlig måte og i tillegg dokumentere i

”Min KOLS bok”.

Prosjektsykepleier skal skrive sykepleiernotat i den Elektroniske Pasientjournal, EPJ ,

etter hjemmebesøk og kontroller.
