# Supplementary material for: Does an Integrated Care Intervention for COPD Patients Have Long-Term Effects on Quality of Life and Patient Activation? A Prospective, Open, Controlled Single-Center Intervention Study
Source: PLoS One. 2017 Jan 6;12(1):e0167887. doi: 10.1371/journal.pone.0167887 (PMC5218408; doi:10.1371/journal.pone.0167887)
Supplement: S4 Text — (DOCX) [file pone.0167887.s004.docx]

**COPD –Home study.**

**English translation of the main points of the protocol.**

**1.1 Introduction**

The project aims to develop a system for home-based treatment, monitoring, care and rehabilitation of patients with severe chronic obstructive pulmonary disease. The project will focus on improved, more active and effective collaboration between professions and levels of health care, that is, between municipal and specialist health services.

**1.2. Background**

Patients with severe COPD and incipient respiratory failure requiring advanced treatment and monitoring. Number of hospitalizations due to COPD was in 1995 over 15000. Most of these (53%) were in the age group over 65 years, an age group that is growing in number. In 1995, the average hospital stay for these patients was 7.9 days, while lying time otherwise for a lung patient was 5.3 days. It must also be noted that these patients are also using 8-12 weeks to recover. It is therefore very important that the basic treatment of this patient population is optimal, and they start with early treatment if they develop symptoms of deterioration.

**1.3. Aims**
The main aim is to improve the home-based treatment of patients with severe lung disease. We suppose that the patients will experience increased levels of mastery of their own situation and an improved quality of life.

**1.4. Hypothesis**

Improved coordination between levels of health care will be of significance to the individual patient's physical and mental health and lead to changes in the patient's needs and consumption of health services. Our hypothesis is that the needs decrease and will lead to health benefits for the individual and economic benefits for health care. Intervention should be compared with a group receiving usual care.

**1.6 Methods**

The study design was a prospective, open, single-centre intervention study.

**Intervention**

All patients hospitalized with AECOPD were treated according to the local and international guidelines*.*

Usual care was offered to the participants allocated to the UC group:

- the standard procedure at discharge from the hospital
- the patient was given written information concerning the disease status and medication.
- the patient’s GP received a discharge summary with a copy to the district home-care health service if the patient was receiving any such services. The GPs followed up the COPD patients and referred the patients to a pulmonary specialist if necessary.

The participants in the UC group were evaluated by a study coordinator (a specialist nurse*) at discharge from TUH and during scheduled visits in their own homes after six-, 12- and 24 month of follow-up.

*The specialist nurses are registered nurses with enhanced competence in respiratory medicine.

Integrated care intervention in accordance with the COPD-Home model was offered to the participants allocated to the IC group. The core elements of the COPD-Home model were:

- a call centre staffed by three specialist nurses for support and communication with patients and home-care nurses, and coordination between the various levels of care. The patients were routinely (at least once a month) contacted by the specialist nurses, and they were supported by telephone calls during COPD exacerbations
- an education session for home-care nurses: a three-hour theoretical session covering several aspects of COPD and two days of practice at the DTM
- an interactive 15-minute e-learning program for the patients concerning the management of COPD
- an individualized self-management plan introduced to the patient at discharge by the treating doctor and a specialist nurse. The plan contains tools for the monitoring of symptoms and written instructions for the self-initiation of prednisone and/or antibiotics, provided specific symptoms have been recorded;
- joint visits at the patients home by the specialist nurse (together with the home-care nurse for participants receiving home services) at approximately three days, 14 days, six months, 12 months and 24 months post-discharge. The major components of these visits were repetition of the core elements of the education program, making necessary changes in the patient’s treatment plans and the reinforcing of specific health behaviors. The patient’s GP was also invited to participate in these visits.

All participants included in the COPD-Home study were free to use all available medical services, including their GPs.

The study protocol contained instruction concerning information given to the patient at discharge and did not include instruction regarding the decision to admit or discharge patients.

**1.13 Outcome measures**

The primary outcomes were:

- number of hospital admissions caused by AECOPD (HA)
- number of in-hospital days (HD) due to AECOPD.

The HA and HD due to AECOPD include admittances where pneumonia was diagnosed during the hospital stay. The HA and HD was assessed in three time periods: during the one year prior to the study enrolment, and during the first- and second year of follow-up

The secondary outcomes were:

- to examine the impact of the COPD-Home IDM intervention on health related QOL (measured by The St. George Respiratory Questionnaire score ), symptoms of anxiety and depression ( measured by the Hospital anxiety (HADS-A) and depression (HADS-D) score ) and degree of the patient activation ( measured by the patient activation measure ( PAM ) during 24 months of follow-up and to assess the degree of association between these outcomes.

Age, gender, forced expiratory volume in one second (FEV1), arterial blood gases, the body mass index (BMI) and baseline data on co-morbidity, medical treatment, lifestyle factors, as well as patient characteristics and disease status after one and two years of follow-up, were collected from the study reports.

**1.14 Ethics**

The study was approved by the Regional Committee for Medical and Health Research Ethics (REC Central) , and the participants gave their written informed consent.
